# Do Experimental Manipulations of Pathogen Avoidance Motivations Influence Conformity?

**DOI:** 10.1177/01461672231160655

**Published:** 2023-03-21

**Authors:** Florian van Leeuwen, Bastian Jaeger, Willem W.A. Sleegers, Michael Bang Petersen

**Affiliations:** 1 Tilburg University, The Netherlands; 2 Aarhus University, Denmark

**Keywords:** pathogen avoidance, disgust, behavioral immune system, conformity, norms

## Abstract

By conforming to ingroup norms, individuals coordinate with other group members, preserve cohesion, and avoid costs of exclusion. Previous experiments have shown that increased concerns about infectious disease increase conformity. However, coordination with other group members has multiple benefits, most of which exist independent of pathogenic infection. Hence, a strong causal effect of pathogen avoidance motivations on conformity seems unlikely. Results from five experiments (*N* = 1,931) showed only limited support for the hypothesis that experimentally increasing pathogen avoidance motivations influences conformity. Overall, our findings are not consistent with the notion that the human mind contains a fast-acting psychological mechanism that regulates conformity as a function of short-term pathogen avoidance motivations.

Infectious disease influences many aspects of human psychology and behavior. There is broad support for the idea that humans have a behavioral immune system, a collection of psychological mechanisms that enables individuals to detect pathogens and motivate behaviors that prevent these pathogens from entering the body (Murray & Schaller, 2016; Tybur & Lieberman, 2016). Ongoing research investigates how the behavioral immune system works; what information it takes as input; what cognitions, emotions, and behaviors it produces as output; and how these outputs influence broader social and cultural phenomena. Here, we test the specific hypothesis that pathogen threats increase conformity (Murray et al., 2011; Murray & Schaller, 2012).

## Explaining the Relation Between Pathogen Avoidance and Conformity

People often conform to norms, especially when they are uncertain about what to do (*informational* conformity), or when they want to fit in or be accepted by their group members (*normative* conformity; Cialdini & Goldstein, 2004). Previous work has developed two candidate explanations for why perceiving infection risks would increase conformity.^1^ One explanation is based on the assumption that throughout human evolution help from ingroup members was crucial for health care (Sugiyama, 2004). People who used normative attitudes to signal ingroup membership when faced with the threat of infectious disease may have received more help than those who did not display normative attitudes. Therefore, there was selection for psychological mechanisms that output normative attitudes (i.e., attitudes expressing normative conformity) contingent on perceiving the threat of infectious disease (Navarrete & Fessler, 2005, 2006). The second explanation assumes that conformity is sometimes an effective strategy to address the problem of infectious disease (Murray et al., 2011; Murray & Schaller, 2012; Schaller, 2016). Humans often cannot perceive pathogens directly and only particular behaviors effectively protect against contamination. Consider, for example, food preparation. Rather than using trial-and-error learning to arrive at a safe way of preparing a particular food, an individual could copy the procedures used by healthy others. Hence, this may constitute an alternative selection pressure for a psychological mechanism that increases conformity contingent upon cues of infection risk.

How would such a psychological mechanism work? Work on this question originated from research on the cross-cultural relation between infectious disease and conformity (Fincher et al., 2008; Gelfand et al., 2011; Schaller & Murray, 2008; Murray et al., 2011). When discussing several candidate explanations for the cross-cultural relation between infectious disease and conformity, Murray and colleagues (2011, p. 327) elaborate on the possibility that a psychological mechanism causes immediate effects of pathogen cues on conformity, proposing that “[i]ndividuals’ behavioral tendencies toward conformity—and their cognitions bearing on those behaviors—vary as a function of perceptual cues indicating the presence of threats in their immediate social context.”

## Empirical Evidence

The most direct evidence for a relation between pathogen avoidance and conformity comes from two kinds of studies: studies of individual differences and psychological experiments.

### Individual Differences

Multiple studies of the relation between the behavioral immune system and conformity have examined individual differences. These studies show that people who are by disposition more motivated to avoid pathogens tend to report stronger traits and attitudes related to conformity to ingroup norms. For example, one study among U.S. citizens showed that individual differences in perceived vulnerability to disease were correlated with ethnocentrism (Navarrete & Fessler, 2006, Study 1). Other studies have reported similar correlations with different measures (Murray & Schaller, 2012; Wu & Chang, 2012).

This association is consistent with patterns of individual differences observed in studies of ideology (Tybur et al., 2016), moral values (van Leeuwen et al., 2017), and personality (i.e., openness to experience; Tybur & de Vries, 2013). Furthermore, a meta-analysis showed that individual differences in pathogen avoidance motivations (such as pathogen disgust sensitivity or germ aversion) were reliably related to social conservatism, a facet of political ideology that relates to conformity to ingroup norms (Terrizzi et al., 2013).

In short, there is substantial evidence that individuals who are by disposition more avoidant of pathogens are also more prone to conform to ingroup norms. However, these studies do not provide good evidence that pathogen avoidance motivations directly cause conformity; they are also consistent with a slower developmental process in which pathogen avoidance motivations influence the development of conformist traits.

### Experimental Evidence

We identified four published experiments that included a manipulation of pathogen avoidance motivations (e.g., showing images of pathogen-relevant stimuli such as feces that evoke pathogen disgust) and a measure of conformity.^2^ In one study, experimentally increased pathogen disgust increased ethnocentrism, as measured by increased liking of an ingroup member who displays a positive opinion about the ingroup (Navarrete & Fessler, 2006, Study 2). In three other experiments, increased pathogen disgust increased self-reported conformity (Wu & Chang, 2012, Study 3), conformity to the group average when evaluating art paintings (Wu & Chang, 2012, Study 2), and behavioral conformity as measured by whether participants followed the majority opinion when casting a vote (Murray & Schaller, 2012).

Each of the experiments has at least one limitation that reduces confidence in its internal validity. In the experiment reported by Navarrete and Fessler (2006), the treatment that increased infection risk involved asking half of the participants to complete a disgust sensitivity scale, whereas the participants in the control condition did not complete this scale. As this study did not include a manipulation check, it is uncertain whether this treatment increased worries about infectious disease or feelings of disgust. While the items of the disgust sensitivity scale likely triggered some pathogen-relevant ideation (e.g., “You are about to drink a glass of milk, when you smell that it is spoiled”), there is a lack of independent evidence that this provided a successful manipulation.

The experiment by Murray and Schaller (2012) included five other measures of conformity in addition to behavioral measure described above. Across the six measures, the findings were not consistent. For the four primary measures, the pathogen threat manipulation (compared with the neutral control) only significantly increased scores for the behavioral measure of conformity. Unfortunately, as the authors discuss, this was also the only measure that could have been influenced by experimenter effects (the experimenter was not blind to the participant’s condition). For the other three primary measures, the effects were not significant. Of the two secondary measures of conformity, only one showed a significant effect of the pathogen threat manipulation. In addition, as the sample size in this study was small (for the primary measures, there were 61–82 participants per condition; for the secondary measures, there were 23–39 participants per condition), these inconsistent effects do not constitute strong evidence.

Finally, the two experiments reported by Wu and Chang (2012, Studies 2 and 3) also involved small samples (less than 35 participants per condition). Taken together, the existing evidence is relatively weak due to limitations of the studies.

## Theoretical Issues

As mentioned above, one explanation for the relation between pathogen avoidance and conformity assumes that conformity is an effective strategy for recruiting social support. While conformity helps buffer against the risk of ostracism (Wesselmann et al., 2014), illness requires high-level investments from others (Sugiyama, 2004) and hence requires a positive attitude rather than the mere absence of a negative attitude. Arguably, positive attitudes of the necessary magnitude require that people believe that you are personally valuable to them, which goes beyond weaker signals of cultural conformity (DeScioli et al., 2011; Tooby & Cosmides, 1996). Second, because gathering social support could take time, a psychological mechanism that upregulates conformity based on moment-to-moment monitoring of infection risk in the local environment might not achieve the optimal level of conformity for gathering social support. Starting to recruit social support when infection risk is imminent may be too late. Third, social support is useful not just for health care, but for dealing with diverse adaptive challenges. Therefore, seeking social support contingent on cues of infection risk might not be an optimal strategy. People tend to cultivate social support continuously, not just when the need for support momentarily arises (Tooby et al., 2006).

The explanation that assumes that conformity is an effective strategy for pathogen avoidance because norms contain precautionary knowledge also suffers from a theoretical issue. While this might apply to some norms (e.g., norms related to food preparation), it is unlikely that this applies to norms in general. For example, some norms about physical contact (e.g., customary handshakes, traditional gatherings) might increase rather than decrease infection risk, whereas other norms (e.g., dress code) seem unrelated to pathogen avoidance. From an optimality perspective, when conformity to norms entails costs and benefits unrelated to pathogens, conformity should not be strongly regulated by cues about pathogen risk.

## Overview of Current Research

We conducted six experiments (all but one was preregistered) in which we manipulated pathogen avoidance motivations and measured conformity. In each experiment, an increase in conformity in the pathogen avoidance condition would count as support for a causal relation between pathogen avoidance motivations and conformity.^3^ Across the six experiments, we recruited samples from different populations, used different measures of conformity, and different manipulations of pathogen avoidance motivations (see Table 1). Prior work suggests that a broad range of manipulations is suitable for increasing pathogen avoidance motivations, such as images of situations with pathogen cues (e.g., Park et al., 2007; Schaller et al., 2010), images that increase state disgust (Wu & Chang, 2012), guided recall of situations with increased infection risk (Murray & Schaller, 2012), and questions-as-treatment procedures that present survey questions that are likely to trigger disgusting thoughts (Navarrete & Fessler, 2006). We used manipulations similar to those in existing work, using disgust-evoking images of pathogen cues (e.g., feces, spoiled food; Studies 1, 4, and 6) and questions that asked participants to imagine contact with people with cues of infectious disease (Studies 2 and 3). We used a guided-recall task in Study 5. We also used measures of conformity that were similar to those used in prior work, with Study 6 being a preregistered close replication of Murray and Schaller (2012). We performed an internal meta-analysis to assess support for the main hypothesis across the studies. Finally, each study also included a measure of individual differences in pathogen avoidance motivations (i.e., pathogen disgust sensitivity; Tybur et al., 2009). We used this variable in additional analyses of individual differences in pathogen avoidance and conformity.

**Table 1.** Overview of Samples, Methods, and Results of Studies 1 to 6

Study	Sample description	Survey language	Sample size *N* (treatment, control)	Manipulation	DV	Treatment condition *M* (*SD*)	Control condition *M* (*SD*)	*t*	*df*	*p*	Cohen’s *d*
1	Dutch-speaking internet users	Dutch	213 (109, 104)	Disgust-inducing images	One of four DVs: • Conformity to loyalty norms • Conformity to hygiene norms • Aversion to loyalty violations • Aversion to hygiene violations	5.41 (0.76) *n* = 32	4.88 (0.96) *n* = 22	2.29	52	.026	0.63
						5.65 (0.69) *n* = 26	5.18 (1.06) *n* = 25	1.87	49	.067	0.52
						4.58 (1.15) *n* = 31	4.30 (1.62) *n* = 24	0.74	53	.464	0.20
						4.95 (1.10) *n* = 20	5.41 (1.20) *n* = 33	−1.40	51	.168	−0.40
2	Students and Dutch-speaking internet users	Dutch or English	509 (256, 253)	Imagined contact	One of four DVs: • Conformity to loyalty norms • Conformity to hygiene norms • Aversion to loyalty violations • Aversion to hygiene violations	4.36 (0.75) *n* = 56	4.61 (0.71) *n* = 79	−1.98	133	.050	−0.35
						5.43 (0.95) *n* = 51	5.66 (0.73) *n* = 49	−1.32	98	.190	−0.26
						4.57 (0.99) *n* = 77	4.84 (0.86) *n* = 59	−1.66	134	.100	−0.29
						5.47 (0.98) *n* = 72	5.57 (0.92) *n* = 66	−0.60	136	.549	−0.10
3	Dutch internet users	English	155 (70, 85)	Imagined contact	Liking of ingroup member that expresses a pro-ingroup attitude	0.82 (1.25) *n* = 64	1.24 (1.49) *n* = 79	−1.77	141	.078	−0.30
4	Students	Dutch or English	276 (138, 138)	Disgust-inducing images	Two DVs: • Conformity to group average when evaluating modern art paintings • Conformity to group norm when this entails infection risk	1.63 (0.61) *n* = 138	1.67 (0.60) *n* = 138	0.50	274	.618	0.06
						2.30 (1.27) *n* = 137	2.12 (1.14) *n* = 137	1.18	272	.240	0.14
5	U.S. residents	English	279 (141, 138)	Guided-recall task	Aggregate index of four measures of conformity	—	—	—	—	—	—
6	U.S. residents	English	790 (389, 401)	Disgust-inducing images	Aggregate index of four measures of conformity	0.032 (0.59) *n* = 389	−0.031 (0.58) *n* = 401	1.51	788	.131	0.11

*Note.* DV = dependent variable.

## Study 1

For all studies reported below, we obtained informed consent from all participants at the start of the survey. The study procedures were approved by the Ethics Review Board of Tilburg School of Social and Behavioral Sciences. All materials, data, and code are available on the Open Science Framework via https://osf.io/84vzp/.

### Method

Study 1 was not preregistered. Participants (*N* = 213) were recruited via social media in January to February 2019 and voluntarily completed an online survey without remuneration.

The study included a between-subjects manipulation of pathogen avoidance motivations using images from the Culpepper Disgust Image Set (Culpepper et al., 2018), which includes images that reliably induce the emotion pathogen disgust and a set of neutral control images. We selected 10 images: Images 1 (dirty sanitary item), 2 (dirty unflushed toilet), 3 (bad dental hygiene), 4 (eating snot bogeys), 11 (decomposing animal carcass), 12 (rotting meat), 13 (liquid rubbish), 14 (stepping in dog feces), 17 (infected wound oozing pus), and 20 (vomit). For the control condition, we used the 10 corresponding pathogen-free images. To encourage participants to view the photos attentively, participants were asked to evaluate each image on a 7-point scale from *very negative* to *very positive*. Participants viewed the photos at their own pace. A measure of state disgust was included as a manipulation check, it was assessed immediately after watching the images (“How much disgust do you experience at the moment?,” rated on an 11-point scale from 0 = *No disgust at all* to 10 = *Very much disgust*). All materials are reported in the Supplementary Materials.

With this between-subjects design, the key comparison is the difference in conformity between the treatment condition (i.e., increased pathogen avoidance motivation) and the control condition. Previous experiments suggest that the effect size for this comparison is in the range of *d* = 0.30 to 0.50. Wu and Chang (2012) reported values of *d* = 0.64 and 0.54. From results reported by Navarrete and Fessler (2006), we estimated *d* = 0.32. From results reported by Murray and Schaller (2012), we estimated *d* = 0.54 for the aggregate conformity index. To achieve power = .80 to detect a significant difference would require *N* = 128 when assuming *d* = 0.5 and *N* = 352 when assuming *d* = 0.3 (for a two-tailed *t* test with α = .05). A sensitivity analysis showed that the current study had power of .80 for a minimum effect size of 0.39 (for the difference between the treatment and the control condition).

The study included novel measures of conformity. We assumed that people may express conformity in at least two different ways, self-presentation (i.e., self-reported norm-following) and aversion toward norm violators. We included measures of both. In addition, we varied the content of the norms being considered: participants completed items either related to ingroup loyalty norms or hygiene norms. Thus, norm-following was measured by asking participants to rate their agreement with statements in which the self was described as a loyal member of their group (12 items, e.g., “I never betray my group,” Cronbach’s α = .88) or as a hygienic person (10 items, e.g., “I never sneeze in the direction of other people,” Cronbach’s α = .84), on a 7-point scale from *strongly disagree* (1) to *strongly agree* (7). Aversion to norm violations was measured by asking participants to evaluate another person of the same sex and age who uttered a statement indicative of a norm violation. Aversion to norm violations was measured with statements about loyalty violations (12 items, e.g., “I often betray my group,” Cronbach’s α = .94) or statements about hygiene violations (10 items, e.g., “I often sneeze in the direction of other people,” Cronbach’s α = .92) all rated on a 7-point scale from *I would not view him or her negatively at all* (1) to *I would view him or her very negatively* (7). Participants were randomly assigned to complete one of the four measures.

The survey included questions to measure sex and age and items from the Three-Domain Disgust Scale (TDDS; Tybur et al., 2009). The TDDS includes seven items that measure pathogen disgust sensitivity, which reflects individual differences in pathogen avoidance motivations. Respondents indicated how disgusting they find the activity described in the item (e.g., “Accidentally touching a person’s bloody cut,” 0 = *not at all disgusting*, 6 = *extremely disgusting*). The survey included additional items unrelated to the current study.

### Results

The manipulation was successful in increasing pathogen avoidance motivations. Participants in the treatment condition reported feeling more disgusted, *M* = 7.09, *SD* = 3.15, than participants in the control condition, *M* = 2.45, *SD* = 1.99, *t*(211) = 12.77, *p* < .001. The effect size for this difference was large, Cohen’s *d* = 1.75.

Next, we estimated the effect of the treatment on conformity. We computed a factorial three-way analysis of variance (ANOVA) including three factors: condition (pathogen avoidance vs. control), type of conformity (adherence to norm vs. aversion to norm violation), and type of norms (loyalty norms vs. hygiene norms). The effect of the pathogen avoidance manipulation was not significant, *F*(1, 205) = 2.31, *p* = .13. The ANOVA also showed a significant Pathogen Avoidance × Type of Conformity interaction, *F*(1, 205) = 4.53, *p* = .035, which indicated that the effect of the manipulation was not equivalent across the conformity measures. There was a significant positive effect of pathogen avoidance on adherence to norms, *M_pathogen_* = 5.53, *M_control_* = 5.03, *t*(205) = 2.32, *p* = .021, Cohen’s *d* = 0.32, but no significant effect on aversion to norm violations, *M_pathogen_* = 4.76, *M_control_* = 4.86, *t*(205) = 0.43, *p* = .67, Cohen’s *d* = 0.06. No other effects were significant. More detailed statistics are reported in the Supplemental Materials.

We examined whether the effect of condition was contingent on controlling for pathogen disgust sensitivity. We included pathogen disgust sensitivity as a covariate, which was significantly related to the conformity measure, *F*(1, 193) = 12.78, *p* < .001. The effect of condition remained not significant, *F*(1, 193) = 3.73, *p* = .055, and the Condition × Type of Conformity interaction remained significant, *F*(1, 193) = 5.22, *p* = .023. There were no other interactions involving the conditions, although there was a significant Type of Norms × Type of Conformity interaction. We then included all interactions of pathogen disgust sensitivity with the factors. This revealed no significant interaction effect between pathogen disgust sensitivity and condition, *F*(1, 186) = 0.51, *p* = .48, indicating that the effect of condition was not moderated by pathogen disgust sensitivity. No effects were significant in this model.

## Study 2

### Method

Study 2 was preregistered (https://aspredicted.org/b5g4f.pdf). Compared with Study 1, Study 2 used a different manipulation of pathogen avoidance motivations and had a larger sample size. In total, 547 participants completed the survey during November 2018 to January 2019. Participants were recruited via social media and the undergraduate research pool at a Dutch university. Participants recruited via social media did not receive remuneration; those participating via the research pool received partial course credit. Participants could select the survey language as Dutch or English. During data processing, a translation error was discovered for one version of the dependent variable (adherence to hygiene norms, English version). Participants who had completed this erroneous dependent variable (*n* = 38) were excluded from the analysis; this exclusion criterion was not specified in advance. The preregistration specified that participants would be excluded if they answered fewer than 75% of the items of the dependent variable; this criterion did not yield any exclusions. Due to miscommunication, we deviated from the preregistered stopping rule (i.e., collect data for 2 weeks and if *N* < 400 collect data for another week) and more participants completed the survey. A sensitivity analysis showed that the sample size provided power of .80 for a minimum effect size of 0.25.

Pathogen avoidance motivations were manipulated by presenting participants with facial photographs of White adult males with or without conspicuous pathogen cues (images were from the Park Aging Mind Laboratory face database; Minear & Park, 2004). In the control condition, unmodified (regular) faces were presented. In the treatment condition, all images had been modified to show a conspicuous cue of infection (e.g., inflamed skin, open sores). All participants viewed eight faces and rated each face for comfort with contact (“How would you feel about shaking hands with the person in the picture?” with 1 = *very uncomfortable*, 5 = *very comfortable*) and health (“How healthy does this person look?” with 1 = *very unhealthy*, 5 = *very healthy*). Participants viewed the photos at their own pace. The manipulation thus involved stimuli that resemble those perceived by the participants in their daily lives when they meet people with or without visible pathogen cues. The questions about contact were included so that participants imagined physical contact with these individuals.^4^ The ratings for health were aggregated across the eight faces and served as a manipulation check. See the Supplementary Materials for details.

Participants completed the same measures of conformity as in Study 1. Reliability of the dependent variables was adequate (Cronbach’s αs ranging from .79 to .86). The survey included items to measure pathogen disgust sensitivity and other items unrelated to the current study.

### Results

The manipulation successfully increased perceived infection risks. Participants in the treatment condition rated the target individuals as less healthy, *M* = 2.31, *SD* = 0.54, compared with participants in the control condition, *M* = 3.20, *SD* = 0.45, *t*(507) = 20.04, *p* < .001. The effect size for this difference was large, Cohen’s *d* = 1.78.

Next, we estimated the effect of the treatment on conformity. Following the preregistration, we computed a factorial three-way ANOVA including three factors: condition (pathogen avoidance vs. control), type of conformity (adherence to norm vs. aversion to norm violation), and type of norms (loyalty norms vs. hygiene norms). The ANOVA showed no significant effect for condition, *F*(1, 501) = 1.65, *p* = .200. The point estimate for conformity was lower in the infection risk condition (*M* = 4.96) than in the control condition (*M* = 5.17). The ANOVA also showed a significant effect for the type of norms, *F*(1, 501) = 43.47, *p* < .001, indicating that conformity was higher for hygiene norms (*M* = 5.53) than for loyalty norms (*M* = 4.60). No other effects were significant. More detailed statistics are reported in the Supplemental Materials.

We examined whether the effect condition was contingent on controlling for pathogen disgust sensitivity. We included pathogen disgust sensitivity as a covariate, *F*(1, 491) = 15.43, *p* < .001. The effect of condition remained nonsignificant. We then included all interactions of pathogen disgust sensitivity with the factors. This revealed no significant interaction effects, indicating that the effect of condition was not moderated by pathogen disgust sensitivity.

As mentioned above, English-speaking participants who completed the dependent variable for adherence to hygiene norms were excluded from the analysis because of a translation error. This may have introduced differences between the participants in the conditions. To account for this, we performed an additional analysis. We computed the three-way factorial ANOVA, but with only including Dutch-speaking participants in all conditions (*N* = 409). The effect of condition remained not significant, *p* = .19.

## Study 3

Studies 1 and 2 used novel measures of conformity. Study 3 tested the effect of pathogen avoidance on conformity with a measure of conformity that had been used in prior research and that seemed suitable for an online study.

### Method

Study 3 was preregistered (https://aspredicted.org/e74dh.pdf). In January to February 2019, 364 participants responded to an online survey. Participants were recruited via social media and received no remuneration. Because of how conformity was measured, the analysis was restricted to Dutch citizens, giving *N* = 155. This exclusion rule was specified in advance. A sensitivity analysis showed that the study had power of .80 for a minimum effect size of 0.46.

Study 3 included the same manipulation of pathogen avoidance motivations as in Study 2, with the perceived health of the targets rated on an 11-point scale from *Very ill* (−5) to *Very healthy* (+5). Study 3 included a measure of conformity similar to that used in the experiment by Navarrete and Fessler (2006). This measure operationalized conformity as adherence to ethnocentric pro-ingroup norms. We included the 7-item measure of ingroup attraction used by Navarrete and Fessler (2006, Study 2). Participants first read a negative opinion about their ingroup (i.e., people of the Netherlands) ostensibly written and posted on the internet by an immigrant. Then participants completed seven items that measured their liking of the author of the anti-ingroup essay (e.g., “How likable is the author of this opinion?” “How intelligent is the author?” all items rated on a 9-point scale from −4 to +4 where −4 reflected a negative evaluation and +4 reflected a positive evaluation). Subsequently, participants read a positive opinion about their ingroup (i.e., people of the Netherlands) ostensibly written and posted on the internet by a Dutch person. Participants then evaluated the author by completing the same seven items. The evaluation of the pro-Dutch author served as the measure of ethnocentrism (Cronbach’s α = .93). This measure of ethnocentrism reflects conformity in the sense that norms typically prescribe expressing positive rather than negative attitudes toward ingroup members. (Given the nature of this measure, the analysis only included participants who indicated Dutch citizenship.)

The survey included items to measure pathogen disgust sensitivity and other items unrelated to the current study.

### Results

The manipulation successfully increased perceived infection risks. Participants in the treatment condition rated the target individuals as less healthy (*M* = −0.90, *SD* = 1.58) compared with participants in the control condition (*M* = 1.17, *SD* = 1.25), *t*(144) = 8.83, *p* < .001. The effect size for this difference was large, Cohen’s *d* = 1.47.

Next, we estimated the effect of the treatment on conformity. We regressed ethnocentrism on condition (0 = control, 1 = infection risk), and following the preregistration, we included sex, age, and pathogen disgust sensitivity as predictors. The effect for condition was not significant, *b* = −0.36, *SE* = 0.23, *t*(128) = −1.55, *p* = .123 (see Supplemental Materials for more detailed statistics). Note that the sign for the point estimate of the effect was negative, indicating an effect in the opposite direction to the hypothesis. We examined whether the effect of condition was moderated by pathogen disgust sensitivity, but the Condition × Pathogen Disgust Sensitivity interaction was not significant, *b* = 0.13, *SE* = 0.14, *t*(127) = 0.92, *p* = .36.

## Study 4

### Method

Study 4 was a preregistered replication of Study 2 by Wu and Chang (2012), but with a larger sample size (http://aspredicted.org/xu8d4.pdf). Two hundred seventy-nine participants completed an online survey in April 2019. Participants were recruited via the undergraduate research pool at a Dutch university and received partial course credit. The preregistration specified exclusion of participants who provided the same response for all items on each of the dependent variables. Following this exclusion rule, two participants were excluded. In addition to this preregistered exclusion rule, a single participant expressed skepticism about one of the dependent variables and was therefore excluded from the analysis, giving a final sample size of *N* = 276. A sensitivity analysis showed that the study had power of .80 for a minimum effect size of 0.34.

The study included a between-subjects manipulation of pathogen avoidance motivations like Study 1. Pathogen avoidance was manipulated by presenting the same images from the Culpepper Disgust Image Set (Culpepper et al., 2018), but with Images 11 and 13 replaced with Images 7 (worms in food) and 8 (dirty fungus infected toenails). To encourage participants to view the photos attentively, participants were instructed to pay attention to the photos because they would have to answer questions about them afterward. Each photo was then presented for at least 4 s after which participants could click a button to proceed to the next image. We used the 10 corresponding pathogen-free images for the control condition. All materials are reported in the Supplementary Materials. The survey included questions measuring state disgust, that is, how disgusted participants felt at that moment, rated on a scale from *Not at all* (1) to *Very much* (7), which served as the manipulation check. State disgust was assessed after participants had completed the first dependent variable.

Participants completed two measures of conformity, one without pathogen cues and one with pathogen cues. The first measure was similar to that used by Wu and Chang (2012). This measure involved participants rating 10 modern art paintings. For each painting participants were shown the average rating of a sample of students from the same university that had participated in a pilot study. The measure of conformity was the absolute difference between the participant’s rating and the average rating shown. For this absolute difference score, lower values indicate higher conformity. Reliability of this measure was sufficient (Cronbach’s α = .73).

The second dependent variable was included to assess whether infection risk manipulation influenced how participants traded off pathogen avoidance versus conformity to an ingroup. Participants were presented with a vignette that asked them to imagine that they were planning to go on vacation with a group of friends and that their friends picked a vacation house to rent. The vignette explained that all other members of the group already had agreed with the choice for the vacation house and that they just wanted to know whether the participant also agreed. The participant was then shown pictures of the vacation house which showed that the house had a dirty bathroom with mold. In this context, conformity with the majority decision—agreement to rent the dirty vacation house—would constitute prioritizing conformity over pathogen avoidance. In contrast, deviance from the majority decision—disagreement with renting the dirty vacation house—would constitute prioritizing pathogen avoidance over conformity. Agreement to rent the dirty vacation house was measured with four items (e.g., “I would certainly approve of the group’s choice for this holiday home,” “I would never accept the choice of this holiday home” [reverse-scored]), rated on a 7-point scale from *strongly disagree* (1) to *strongly agree* (7), Cronbach’s α = .87. The survey also included items to measure pathogen disgust sensitivity.

### Results

The manipulation successfully increased pathogen avoidance motivations. Participants in the treatment condition (*M* = 4.37, *SD* = 1.73) reported feeling more disgusted than participants in the control condition (*M* = 2.13, *SD* = 2.13), *t*(274) = 12.11, *p* < .001. The effect size for this difference was large, Cohen’s *d* = 1.46.

Next, we estimated the effect of the treatment on conformity, following the preregistration. A *t* test revealed no significant difference in deviance between the control condition (*M* = 1.67, *SD* = 0.60) and the infection risk condition (*M* = 1.63, *SD* = 0.61), *t*(274) = 0.50, *p* = .618, *d* = 0.06 (see Table 1). Similarly, a *t* test revealed no significant difference in conformity to the vacation group between the control condition (*M* = 2.12, *SD* = 1.14) and the infection risk condition (*M* = 2.30, *SD* = 1.27), *t*(272) = 1.18, *p* = .240, *d* = 0.14.

To control for individual difference variables, we regressed the deviance score on condition (0 = control, 1 = infection risk), age, sex, and pathogen disgust sensitivity. The effect of condition remained not significant, *b* = −0.04, *SE* = 0.07, *t*(267) = −0.55, *p* = .58. We also regressed conformity to the vacation group on condition, age, sex, and pathogen disgust sensitivity. The effect of condition remained not significant, *b* = 0.14, *SE* = 0.14, *t*(266) = 0.96, *p* = .34 (see Supplemental Materials for more detailed statistics).

## Study 5

Study 5 was designed as a close replication of the experiment reported in Murray and Schaller (2012), arguably the best existing experiment on the relation between pathogen avoidance and conformity. Study 5 was preregistered (https://aspredicted.org/xr4sr.pdf). We consulted with the first author of the original study to verify that our materials and procedures resembled those of the original study. The original study manipulated pathogen avoidance motivations via a guided-recall procedure that was administered by the experimenter in a face-to-face interview. When we collected data for this study (May 2021), it was not feasible to do face-to-face interviews with a large sample of participants due to COVID-19 restrictions. Therefore, we adjusted the materials to an online survey and collected data from U.S. residents via Prolific who completed the study for a payment of 1.50 GBP. Because there was uncertainty whether the manipulation would be effective in an online survey, we preregistered to collect the data in batches. In Batch 1, we collected data from 280 participants, for which we evaluated whether the pathogen avoidance manipulation was successful at (a) increasing state disgust compared with the control condition, and (b) inducing disgust, but not fear. We included this second criterion because in the original study, the manipulation was designed to be specific to disgust (i.e., it involved recall of disgusting situations more so than fear-evoking situations). With 280 participants, the power for each of these tests (assuming *d* = 0.3) is .80 and .99, respectively. If the manipulation check were to indicate successful manipulation of disgust, then we would collect the remaining participants in a second batch.

Unfortunately, the manipulation checks indicated that the manipulation did not work as intended. The manipulation increased state disgust in the treatment condition (*M* = 2.79, *SD* = 1.78) compared with the control condition, *M* = 1.37, *SD* = 0.98, *t*(277) = 8.24, *p* < .001, Cohen’s *d* = 0.99. However, the manipulation did not specifically increase state disgust. In the treatment condition, state disgust was not substantially higher (i.e., with *p* < .05 and an effect size of at least 0.3) than state fear, *M* = 2.45, *SD* = 1.60, *t*(140) = 2.71, *p* = .0076, Cohen’s *d* = 0.20. Following the preregistration, we stopped data collection. See the Supplementary Materials for a more detailed description of the methods and results.

## Study 6

Study 6 was similar to Study 5 and was designed as a replication the experiment reported in Murray and Schaller (2012), but with a different manipulation of pathogen avoidance motivations. Study 6 was preregistered (https://aspredicted.org/eu5vv.pdf).

### Method

#### Procedure

To manipulate pathogen avoidance motivations, we used images from the Culpepper Disgust Image Set (Culpepper et al., 2018) as in Studies 1 and 4. In the pathogen threat condition, participants were shown Images 2, 3, 6, 7, 11, 12, 14, 15, 17, and 20 from the set. In the control condition, we used the corresponding pathogen-free images.

We used the same dependent variables as in Study 5. Participants completed four measures of conformity, three of which—self-reported conformist attitudes (six items), liking for people with conformist traits (three items), valuation of obedience (one item)—were the same as those used by Murray and Schaller (2012). The fourth measure was similar but not identical to the behavioral measure used by Murray and Schaller (2012). Originally, the behavioral measure asked participants to indicate their agreement or disagreement with a proposal about an education policy at their university. Conformity was operationalized as expressing the majority opinion. To adjust this behavioral measure to an online context, we asked people whether they agreed or disagreed with the statement “The National Science Foundation should increase its budget for research of the biodiversity in Antarctica.” The instructions mentioned that in a survey 1 year prior we had also asked about this issue and that this previous survey indicated that a majority agreed (or disagreed, randomized across participants) with the statement. Conformity was operationalized as selecting the majority opinion. The order of the four measures of conformity was randomized across participants. To test the hypothesis, we followed the original study and computed an aggregate index of these four measures by standardizing each measure and then computing the mean score.

Participants then rated the emotions they experienced during the experimental session on 7-point scales from *None at all* (1) to *Extremely* (7); this included a measure of state disgust that served as a manipulation check. Finally, participants reported their political orientation and completed the seven items of the pathogen disgust domain of the TDDS. The survey also included an attention check. Immediately after the last item for pathogen disgust sensitivity, participants were shown the item *Please select “not at all disgusting*,” with the same seven answer options as for the TDDS. Participants who selected any other answer than “not at all disgusting” were considered to have failed the attention check. Finally, participants were thanked and debriefed.

#### Participants

We recruited 790 participants (389 participants in the treatment condition, 401 participants in the control condition) who met the inclusion criteria in June 2021. Assuming a population effect size of *d* = 0.20 (which is smaller than that observed in the original study), the required sample size for a two-sided *t* test for independent means with 80% power is at least 788 (394 per condition). This sample size would also allow for a small telescopes analysis (Simonsohn, 2015), which in this case would require a total sample of 358. We recruited participants via Prolific from the U.S. population, which is culturally similar to the Canadian population sampled by Murray and Schaller (2012). Hence, differences between this study and Murray and Schaller (2012) cannot easily be attributed to cultural differences between the samples. Participants completed the study for a payment of 1.00 GBP.

### Results

The manipulation successfully increased pathogen avoidance motivations. Participants reported significantly higher state disgust in the treatment condition (*M* = 5.39, *SD* = 1.78) than in the control condition (*M* = 1.94, *SD* = 1.22), *t*(788) = 31.84, *p* < .001, Cohen’s *d* = 2.27. Participants in the treatment condition reported significantly higher state disgust than state fear (*M* = 2.09, *SD* = 1.57), *t*(388) = 34.06, *p* < .001, Cohen’s *d* = 1.96. In addition, state fear was significantly higher in the treatment condition than in the control condition, *M* = 1.28, *SD* = 0.84, *t*(788) = 9.09, *p* < .001, Cohen’s *d* = 0.64.

Next, we estimated the effect of the treatment on conformity. Scores for the aggregate index of conformity were not significantly higher in the treatment condition than in the neutral control condition, *t*(788) = 1.51, *p* = .131, *d* = 0.11 (see Table 1 for means). We evaluated the robustness of these results. We tested the hypothesis over the full sample (i.e., an intention-to-treat analysis, including all participants who completed the dependent variables) with a two-sided *t* test. The difference remained not significant, *t*(789) = 1.44, *p* = .15. To explore whether the effect was specific to a particular measure of conformity, we performed a *t* test for each of the four measures of conformity (i.e., four two-sided *t* tests with *p* values corrected for multiple comparisons with the Holm–Bonferroni method). This showed that the manipulation did not influence the scores for self-reported conformist attitudes, *t*(788) = 0.71, *p* = 1.00; liking for people with conformist traits; *t*(788) = 0.72, *p* = 1.00; valuation of obedience, *t*(788) = 1.76, *p* = .31; and siding with the majority opinion, *b* = 0.05, *SE* = 0.15, *z*(788) = 0.33, *p* = 1.00.

## Internal Meta-Analysis

**Figure 1.**
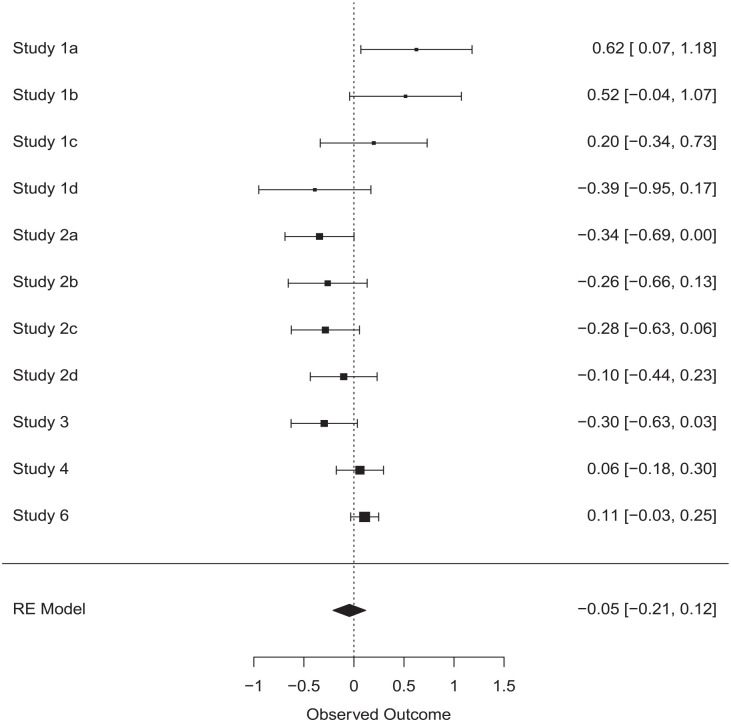
Forest Plot for the Meta-Analysis of Effects of the Infection Risk Manipulation on Conformity RE Model = Random Effects Model. *Note.* The effect sizes shown are Cohen’s *d*.

We performed an internal meta-analysis to combine the evidence from our experiments. We performed a random-effects meta-analysis over Cohen’s *d* effect sizes reported in Table 1. For Studies 1 and 2, we included the effect sizes for each of the four measures of conformity (each participant only completed one measure). For Study 4, we included the effect size for the primary dependent variable, the deviance in art ratings. The average effect across the 11 effect sizes was not significantly different from zero, average *d* = −0.05, 95% confidence interval (CI) = [−0.21, 0.12]. See Figure 1 for the forest plot.

We also performed a Bayesian meta-analysis based on *t* statistics (Rouder & Morey, 2011) using the BayesFactor package (Morey & Rouder, 2018). We performed a *t* test in each of the 11 comparisons testing the difference between the control and treatment condition on the main conformity measure. The resulting *t* statistics and condition sample sizes were entered into the meta-analysis, resulting in a Bayes factor of 19.16 in favor of the null hypothesis. This constitutes strong evidence for the absence of an effect (Jeffreys, 1998).

In addition, we performed a random-effects meta-analysis across our studies and the four prior experiments that tested for an effect of pathogen avoidance motivations on conformity (see the section “Experimental Evidence”). This estimated an average effect size of *d* = 0.09 that was not significantly different from zero, 95% CI = [−0.07, 0.26].

Finally, we explored whether the effect of pathogen avoidance motivations on conformity was moderated by (a) participant’s level of disgust sensitivity, (b) participant gender, (c) the type of manipulation (viewing disgust inducing images vs. imagined physical contact with a person with facial blemishes), (d) the type of dependent variable (self-report measure of conformist traits vs. other). We aggregated the data from all studies and estimated a regression model in which conformity (*z*-standardized within each study) was regressed on condition, disgust sensitivity, gender, manipulation type, dependent variable, and the interactions between condition and the four candidate moderator variables. Examining the interaction effects showed that the effect of condition on conformity was not significantly moderated by disgust sensitivity, *b* = −0.016, *SE* = 0.047, *t*(1,738) = −0.34, *p* = .732; gender, *b* = −0.088, *SE* = 0.102, *t*(1,738) = −0.87, *p* = .385; or the type of dependent variable that was used, *b* = −0.220, *SE* = 0.150, *t*(1,738) = −1.46, *p* = .143. However, we did find a significant interaction effect between condition and manipulation type, *b* = −0.579, *SE* = 0.165, *t*(1,738) = −3.51, *p* < .001. Participants in the pathogen condition scored higher on conformity than participants in the control condition when pathogen avoidance was manipulated by having participants view disgusting images from the Culpepper Disgust Image Set, *b* = 0.201, *SE* = 0.076, *t*(1,738) = 2.65, *p* = .008. Because the outcome measures were standardized, the regression coefficient can be interpreted as an effect size, indicating that the effect was small (i.e., *d* = 0.20). In contrast, when participants imagined physical contact with a person displaying facial blemishes, the effect was in the *opposite* direction, with participants in the control condition showing higher levels of conformity than participants in the pathogen condition, *b* = −0.378, *SE* = 0.120, *t*(1,738) = −3.16, *p* = .002.

## Additional Analysis

Each of the studies included a measure of pathogen disgust sensitivity. Table 2 shows the correlations between pathogen disgust sensitivity and the outcome variables for all studies. In Study 1, we found significant positive correlations between disgust sensitivity and three of the four measures of conformity (the correlation was not significant for conformity to hygiene norms). In Study 2, we again found significant positive correlations for three of the four measures (the correlation was not significant for conformity to loyalty norms). In Study 3, the correlation between disgust sensitivity and ethnocentrism was not significant. In Study 4, the correlation with disgust sensitivity was not significant for the primary measure (art ratings), and was significant, but negative, for the secondary measure (conformity to the vacation group). In Study 5, disgust sensitivity was significantly correlated with self-reported conformist tendencies, but not with the three other measures. Finally, in Study 6, the correlation with disgust sensitivity was positive and significant for self-reported conformist attitudes and valuation of obedience.

**Table 2.** Correlations Between Pathogen Disgust Sensitivity and Conformity Measures Across the Studies

Study	Dependent variable	*N*	*r*	*p*
Study 1	Conformity loyalty	52	.30	.032
	Conformity hygiene	51	−.06	.661
	Aversion loyalty	50	.34	.015
	Aversion hygiene	49	.33	.021
Study 2	Conformity loyalty	133	.05	.549
	Conformity hygiene	99	.28	.004
	Aversion loyalty	133	.22	.012
	Aversion hygiene	135	.19	.024
Study 3	Ethnocentrism	134	−.02	.815
Study 4	Art ratings (deviance)	275	.004	.944
	Vacation home	274	−.17	.006
Study 5	Conformist attitudes	279	.16	.009
	Liking for people with conformist traits	279	.01	.827
	Valuation of obedience	279	.05	.408
	Agreement with majority opinion	279	−.09	.117
	Aggregate conformity index	279	.06	.358
Study 6	Conformist attitudes	790	.25	<.001
	Liking for people with conformist traits	790	.01	.738
	Valuation of obedience	790	.10	.003
	Agreement with majority opinion	790	−.06	.091
	Aggregate conformity index	790	.13	<.001

Next, we performed an internal meta-analysis of Fisher *z* transformed correlations. For Study 4, the correlation for deviance in art ratings was included (we reversed the sign for this correlation). For Studies 5 and 6, we included the aggregate conformity index across the four measures. The average correlation across all studies, including Study 5, was *r* = .13, 95% CI = [.06, .20].

## General Discussion

Five experiments estimated the effect of pathogen avoidance motivations on conformity. The internal meta-analysis showed that across these experiments, there was no significant effect of pathogen avoidance motivations on conformity. The discrepancy between the current results and those of previous experiments cannot be easily attributed to methodological differences or cultural differences between the sampled populations. Compared with previous studies, our studies typically had larger samples, and Study 6 arguably is the most powerful test of the hypothesis to date. A Bayesian analysis of the aggregate data across all studies showed strong evidence in favor of the null hypothesis, which also suggests that the obtained null result cannot be attributed to low statistical power to detect a meaningful effect. To achieve large samples, our studies were implemented as online surveys, but so was one of the directly relevant existing experiments (Navarrete & Fessler, 2006). We tested the hypothesis with samples recruited from Dutch and U.S. populations, in line with the previous work showing that the effect is observed across U.S., Canadian, and Chinese populations. Previous experiments used various measures of conformity, including measures of ethnocentrism (Navarrete & Fessler, 2006), attitudes toward art paintings (Wu & Chang, 2012), self-reported conformist attitudes (Murray & Schaller, 2012; Wu & Chang, 2012), and perceived severity of infection-relevant normative transgressions (Murray & Schaller, 2012). The current studies included similar measures of conformity.

Previous experiments observed medium-sized effects while using various manipulations, including questions-as-treatment designs (e.g., participants in the treatment condition were asked questions that evoke pathogen-related thoughts or memories of being vulnerable to infectious disease, Murray & Schaller, 2012; Navarrete & Fessler, 2006) and treatments that exposed participants to visual pathogen cues (e.g., images of maggots and wounds, or a video clip about a pandemic, Wu & Chang, 2012). Across the current studies, we used three kinds of manipulations similar to those used in previous experiments: Studies 1, 4, and 6 used validated images with visual pathogen cues; Studies 2 and 3 used a questions-as-treatment procedure to evoke imagined contact with an infectious individual. In Study 5, we attempted a close replication of the guided-recall procedure used by Murray and Schaller (2012) but did not obtain the desired manipulation of pathogen avoidance motivations. Importantly, manipulation checks suggested that the manipulations in Studies 1, 2, 3, 4, and 6 were large enough that an effect on conformity could have been observed.

An exploratory moderator analysis indicated that the effect of the pathogen manipulation was contingent on the type of manipulation used: On average, when the pathogen manipulation involved viewing disgusting images from the Culpepper Disgust Image Set, there was a small increase in conformity, but when the pathogen manipulation involved imagined contact with individuals that display pathogen cues (manipulated via face images), there was a small *decrease* in conformity. Although we found the previously observed effect when using one type of manipulation, this result is not easily reconciled with past studies. None of the previous studies that found a significant effect on conformity used this manipulation and there is no obvious explanation for the fact that the other manipulation (which successfully manipulated perceive infection risk in our studies) led to the opposite effect. As the analysis was exploratory in nature and we tested four plausible moderators in total (increasing the risk of a false-positive effect), we urge caution when interpreting this result. More work is needed to test whether manipulating pathogen avoidance motivations with the Culpepper Disgust Image Set reliably increases conformity by a nontrivial degree, and why other manipulations with pathogen cues would show the opposite effect.

In sum, the current findings provide limited evidence that increased pathogen avoidance motivations have an immediate effect on conformity. In Study 1, the pathogen avoidance manipulation increased conformity, but only when conformity was operationalized as self-reported adherence to norms. However, none of the preregistered studies showed a significant effect consistent with prior experiments and the internal meta-analysis indicated that average effect size was not significantly different from zero. Furthermore, an exploratory moderator analysis showed that the effect of the manipulation was small and in the predicted direction, but only when the manipulation used disgusting images (and opposite when it used images of faces with pathogen cues). The combination of our observation of limited evidence while using larger samples than prior work, plus the observation in prior experiments of effects across different manipulations (Murray & Schaller, 2012; Navarrete & Fessler, 2006; Wu & Chang, 2012), and the methodological limitations of those prior experiments (see section “Experimental Evidence,” above), suggests that there is currently only limited supporting evidence for a facultative or perceptual mechanism that increases conformity to ingroup norms contingent on moment-to-moment fluctuations in pathogen avoidance motivations.

In contrast, the current studies did reliably show an association between pathogen disgust sensitivity and conformity. This association is consistent with previous research that showed associations between pathogen disgust sensitivity and traits related to conformity, such as traditionalism (Tybur et al., 2016), openness to experience (Tybur & de Vries, 2013), and social conservatism (Terrizzi et al., 2013). Some research has assumed that states (e.g., feeling pathogen disgust) and conceptually related traits (e.g., being highly pathogen disgust sensitive) have similar relations with outcome variables (e.g., Horberg et al., 2009; Landy & Goodwin, 2015; Makhanova et al., 2019; Maner et al., 2005; Navarrete & Fessler, 2006; Park et al., 2007). To further explore this, we examined the correlations between self-reports of state disgust that served as manipulation checks and conformity in Studies 1, 4, and 6. State disgust was not significantly correlated with conformity, Study 1: *r* = .09, *p* = .179; Study 4: *r* = .02, *p* = .721; Study 6: *r* = −.02, *p* = .518. Based on the current findings, it seems that the relation between pathogen avoidance and conformity is a case where there is a dissociation between effects observed for experimental manipulations (limited evidence for a relation) and patterns of individual differences (strong evidence for a relation).

The current studies do not allow us to pinpoint why this discrepancy between effects for state and trait disgust exists. It is possible that the relation between pathogen avoidance and conformity observed in individual difference studies reflects a common-method bias, the influence of a confounding variable, or a reversed causal path. It is also possible that the relation between pathogen avoidance and conformity is consistent with a developmental process, so that individuals who invest more in pathogen avoidance also develop stronger conformist traits (e.g., Murray et al., 2011; Tybur et al., 2016). A starting point for investigating these developmental processes is work that has examined why people differ in their investment in pathogen avoidance (i.e., why individuals differ in pathogen disgust sensitivity; Tybur et al., 2018). One proposal is that pathogen disgust sensitivity derives from a more general predisposition toward risk-taking (Sparks et al., 2018). Deviating from norms is often risky, so general predispositions for risk-taking may influence pathogen disgust sensitivity and, in turn, conformity. (It is also possible that risk-taking serves as a third variable influencing both disgust sensitivity and risk-taking.) However, some evidence speaks against this proposal. Neuroticism (a broad personality dimension including traits associated with risk avoidance such as fearfulness and anxiety) relates only weakly with disgust sensitivity (Tybur et al., 2018). Further research might consider using longitudinal studies to examine the relation between pathogen disgust sensitivity and conformity.

### Limitations and Future Directions

The current studies have limitations that should be taken into account when interpreting the results. First, across the current studies, we used different manipulations of pathogen avoidance motivations. These manipulations resembled those in prior work. However, while prior work has used a variety of manipulations, it is not well understood what kind of procedures or stimuli are best for manipulating pathogen avoidance motivations (Tybur et al., 2014). For example, it is uncertain whether tests of hypotheses about the effects of pathogen avoidance motivations are best tested with manipulations that induce high levels of pathogen disgust by presenting visual images (Culpepper et al., 2018), by exposure to disgusting smell (Landy & Goodwin, 2015), or with questions-as-treatment designs (Murray & Schaller, 2012). It is possible that what constitutes an ideal manipulation of pathogen avoidance motivations depends on the outcome variable, for example, with some outcomes being sensitive only to manipulations of affect (i.e., the subjective experience of feeling disgusted) and others being sensitive only to manipulations of cognitive components (e.g., worries about infection risk; for discussion see Schaller, 2014). Validated manipulations of pathogen avoidance motivations and theoretical specification of what kind of manipulations should influence what kind of outcomes would be valuable for future research investigating the workings of the behavioral immune system and its influence on cognition and behavior. More generally, our manipulations related to pathogen threat and hence our results do not warrant inferences about the effects of other threats on conformity. Further research may examine the relations between other threats and conformity.

Second, the current studies used several measures of conformity similar to those used in previous research, but these were not “gold standard” measures of behavioral conformity. Based on previous research on the link between pathogen avoidance and conformity, there seems to be no consensus on what operationalization of conformity (e.g., behavioral conformity, conformist attitudes, self-reported conformist behaviors) would most likely be influenced by pathogen avoidance motivations. In Study 3, we operationalized conformity as ethnocentrism, specifically as expressing ethnocentric pro-ingroup attitudes, following research by Navarrete and Fessler (2006). As argued by Navarrete and Fessler (2005), norms of favoring and aiding ingroup members are common across cultures, so that expressing pro-ingroup attitudes is a manifestation of normative conformity. Furthermore, Wu and Chang (2012) interpreted the findings for the ethnocentrism measure used by Navarrete and Fessler (2006) as supporting evidence. However, the measure used in Study 3 is not a direct measure of conformity, has not been validated, and could capture other variables than conformity. Hence, the results for Study 3 should be interpreted with caution. In Study 4, we included a measure of conformity that was designed to measure conformity in a situation that involved infection risk. As a reviewer pointed out, the inclusion of pathogen cues in the measure might have eliminated any effect of the pathogen avoidance manipulation, as all participants—also those in the control condition—were shown salient pathogen cues. This means that the absence of a significant effect for the secondary dependent variable in Study 4 should be interpreted carefully.

More broadly, this suggests that it might be difficult to test whether the effects of pathogen avoidance motivations on conformity are domain-specific, in the sense of occurring especially in contexts with salient infection risk. The most commonly used measure of behavioral conformity is probably Asch’s (1955) line-judgment paradigm. We are not aware of any line-judgment studies that also involved a disgust induction. Further research might explore such behavioral measures, though it might be challenging to combine this method with the sample size required to detect small effects. Future studies could also focus on developing a valid measure of conformity, which is currently lacking. When examining the different measures used here and in previous work, it is unclear to what extent different measures reflect the same underlying construct (e.g., to what extent are self-reported conformist behaviors and conformist behaviors in lab paradigms correlated) and to what extent conformist behaviors in different domains converge (e.g., art judgments vs. loyalty in intergroup conflict). Validated measures of conformity suitable for laboratory and online studies would be valuable, not only for future work examining the link with pathogen avoidance motivations.

Finally, future studies should also rely on more diverse samples of participants to explore whether the current findings generalize to other populations. Participant samples in Studies 1 to 4 mostly consisted of Dutch university students. Participants were relatively young and predominantly female. In Studies 5 and 6, we recruited more diverse samples of U.S. residents from Prolific. It is plausible that results may differ across cultures and it should be noted that the current findings are based on participant samples from two relatively rich, Western countries.

### Implications

The current findings provide limited support for the notion that the behavioral immune system has design features that immediately upregulate conformity contingent on salient pathogen cues. In particular, our findings suggest that effects of pathogen avoidance on conformity are smaller than observed in prior work and might occur only for particular types of manipulations. The primary implication is that further research is needed to establish if and under what conditions pathogen cues increase conformity.

## Supplemental Material

sj-docx-1-psp-10.1177_01461672231160655 – Supplemental material for Do Experimental Manipulations of Pathogen Avoidance Motivations Influence Conformity? Supplemental material, sj-docx-1-psp-10.1177_01461672231160655 for Do Experimental Manipulations of Pathogen Avoidance Motivations Influence Conformity? by Florian van Leeuwen, Bastian Jaeger, Willem W.A. Sleegers and Michael Bang Petersen in Personality and Social Psychology Bulletin

## Notes

1. A third candidate explanation could be derived from terror management theory (TMT; Burke et al., 2010; Renkema et al., 2008). According to TMT, threats to the self should increase normative behavior. If one assumes that a salient infection risk is a threat to the self, then TMT generates the prediction that infection risk should increase conformity. However, work on TMT seems not to have proposed such an explanation, not to have considered pathogens as threats to the self, and it seems there are no TMT studies that have included a manipulation of pathogen threat (Burke et al., 2010). Although asking people to think of their own death might lead to some pathogen-relevant ideation (e.g., decaying corpses, worms), TMT theorists seem to not have thought that the pathogen aspect or its associated emotion (disgust) is driving the effects of mortality salience. Therefore, we do not elaborate on this possible explanation.

2. The 2014 Ebola outbreak has been analyzed as a natural experiment of the effect of pathogen threat on political and social attitudes (Beall et al., 2016). Unfortunately, the results for conformity were equivocal. While some analyses revealed a bandwagon effect (i.e., conformity to local voting norms), other analyses provided no support for this (Schaller et al., 2017; Tiokhin & Hruschka, 2017).

3. Studies 1 and 2 included novel measures of conformity to test hypotheses that distinguished between the two different explanations for the relation between pathogen avoidance and conformity. In short, the social support explanation predicts that the effect of pathogen avoidance on conformity should be stronger for a measure of conformity that captures self-presentation as a loyal member of the ingroup, than for a measure of conformity that captures aversion to violations of hygiene norms. In contrast, the explanation that assumes that conformity protects against infection would make the opposite prediction. However, these hypotheses involved three-way interactions and in retrospect both studies were underpowered for testing these interactions (cf. Blake & Gangestad, 2020). Therefore, the article is focused on the more basic question of whether pathogen avoidance manipulations influence conformity.

4. In contrast with the manipulation used in Study 1, this procedure is not aimed at inducing high levels of disgust. Because feeling disgusted is unpleasant, manipulations that induce high levels of disgust might cause some participants to disengage with the stimuli, reduce compliance, or trigger reactance (e.g., trying to respond as if not feeling disgusted). To address these possible limitations of disgust inductions, we developed these alternative manipulations.
