# Supplementary material for: Do Experimental Manipulations of Pathogen Avoidance Motivations Influence Conformity?
Source: Pers Soc Psychol Bull. 2023 Mar 21;50(7):1051–65. doi: 10.1177/01461672231160655 (PMC11143762; doi:10.1177/01461672231160655)
Supplement: sj-docx-1-psp-10.1177_01461672231160655 – Supplemental material for Do Experimental Manipulations of Pathogen Avoidance Motivations Influence Conformity? [file sj-docx-1-psp-10.1177_01461672231160655.docx]

**Supplementary Materials**

# Do Experimental Manipulations of Pathogen Avoidance Motivations Influence Conformity?

Contents:

Supplementary Analysis

Supplementary Materials Study 1

Supplementary Materials Study 2

Supplementary Materials Study 3

Supplementary Materials Study 4

Supplementary Materials Study 5

# **Supplementary Analysis**

Table S1: ANOVA results (Study 1).

|  | *F* | *p* |
| --- | --- | --- |
| Pathogen avoidance | 2.31 | .130 |
| Norm type | 0.64 | .425 |
| Conformity type | 0.89 | .348 |
| Pathogen avoidance × Norm type | 4.53 | .035 |
| Pathogen avoidance × Conformity type | 0.03 | .873 |
| Norm type × Conformity type | 3.49 | .063 |
| Pathogen avoidance × Norm type × Conformity type | 1.21 | .273 |

Table S2: ANOVA results, when controlling for disgust sensitivity (Study 1).

|  | *F* | *p* |
| --- | --- | --- |
| Pathogen avoidance | 3.73 | .055 |
| Norm type | 1.67 | .197 |
| Conformity type | 0.54 | .465 |
| Pathogen avoidance × Norm type | 5.22 | .023 |
| Pathogen avoidance × Conformity type | 0.01 | .913 |
| Norm type × Conformity type | 4.58 | .034 |
| Pathogen avoidance × Norm type × Conformity type | 2.14 | .146 |
| Pathogen disgust sensitivity | 12.78 | < .001 |

Table S3: ANOVA results (Study 2).

|  | *F* | *p* |
| --- | --- | --- |
| Pathogen avoidance | 1.65 | .200 |
| Norm type | 0.29 | .588 |
| Conformity type | 43.47 | < .001 |
| Pathogen avoidance × Norm type | 0.31 | .581 |
| Pathogen avoidance × Conformity type | 0.14 | .906 |
| Norm type × Conformity type | 2.04 | .153 |
| Pathogen avoidance × Norm type × Conformity type | 0.21 | .650 |

Table S4: ANOVA results, when controlling for disgust sensitivity (Study 2).

|  | *F* | *p* |
| --- | --- | --- |
| Pathogen avoidance | 0.44 | .505 |
| Norm type | 0.10 | .750 |
| Conformity type | 33.23 | < .001 |
| Pathogen avoidance × Norm type | 0.02 | .901 |
| Pathogen avoidance × Conformity type | 0.46 | .496 |
| Norm type × Conformity type | 0.30 | .581 |
| Pathogen avoidance × Norm type × Conformity type | 0.03 | .867 |
| Pathogen disgust sensitivity | 15.43 | < .001 |

Table S5: ANOVA results, when only including Dutch-speaking participants (Study 2).

|  | *F* | *p* |
| --- | --- | --- |
| Pathogen avoidance | 1.71 | .192 |
| Norm type | 2.75 | .098 |
| Conformity type | 46.65 | < .001 |
| Pathogen avoidance × Norm type | 1.23 | .268 |
| Pathogen avoidance × Conformity type | 0.30 | .584 |
| Norm type × Conformity type | 8.03 | .005 |
| Pathogen avoidance × Norm type × Conformity type | 1.37 | .242 |

Table S6: Results of regression analysis (Study 3).

|  | *b* | *t* | *p* |
| --- | --- | --- | --- |
| Pathogen avoidance | -0.356 | -1.55 | .123 |
| Male | -0.472 | -2.06 | .042 |
| Age | -0.031 | -3.01 | .003 |
| Pathogen disgust sensitivity | 0.033 | 0.44 | .663 |

Table S7: Results of regression analysis with interaction effect (Study 3).

|  | *b* | *t* | *p* |
| --- | --- | --- | --- |
| Pathogen avoidance | -1.276 | -1.24 | .217 |
| Male | -0.482 | -2.10 | .038 |
| Age | -0.030 | -2.90 | .004 |
| Pathogen disgust sensitivity | -0.06 | -0.27 | .790 |
| Pathogen avoidance × Pathogen disgust sensitivity | 0.132 | 0.92 | .361 |

Table S8: Results of regression analysis for Art ratings (deviance scores) (Study 4).

|  | *b* | *t* | *p* |
| --- | --- | --- | --- |
| Pathogen avoidance | -0.040 | -0.55 | .580 |
| Male | 0.205 | 2.37 | .019 |
| Age | 0.046 | 2.31 | .022 |
| Pathogen disgust sensitivity | 0.038 | 0.88 | .379 |

Table S9: Results of regression analysis for Conformity to the vacation group (Study 4).

|  | *b* | *t* | *p* |
| --- | --- | --- | --- |
| Pathogen avoidance | 0.139 | 0.96 | .339 |
| Male | 0.160 | 0.93 | .354 |
| Age | -0.072 | -1.82 | .070 |
| Pathogen disgust sensitivity | -0.223 | -2.60 | .010 |

**Results Study 5**

For the sake of completeness, we report the results of the pathogen avoidance manipulation on conformity here. A *t*-test revealed no significant difference in aggregate conformity scores (averaged across the four conformity measures) between the control condition (*M* = -0.017, *SD* = 0.562) and the disease threat condition (*M* = 0.016, *SD* = 0.578), *t*(277) = 0.48, *p* = .632, *d* = 0.06. We found similar results when running separate analyses for the four conformity measures. There was no significant difference in self-reported conformist attitudes between the control condition (*M* = 3.57, *SD* = 1.25) and the disease threat condition (*M* = 3.67, *SD* = 1.28), *t*(277) = -0.64, *p* = .525, *d* = -0.08. There was also no significant difference in liking for people with conformist traits between the control condition (*M* = 3.26, *SD* = 1.05) and the disease threat condition (*M* = 3.16, *SD* = 1.03), *t*(277) = 0.85, *p* = .399, *d* = 0.10. There was also no significant difference in valuation of obedience between the control condition (*M* = 6.01, *SD* = 5.68) and the disease threat condition (*M* = 7.14, *SD* = 7.06), *t*(277) = 1.47, *p* = .143, *d* = -0.18. A chi-square test revealed no significant difference in the number of participants agreeing with the majority opinion between the control condition (73.9%) and the disease threat condition (73.0%), *χ^2^*(1) < .01, *p* = .978. Finally, as the pathogen avoidance manipulation influenced both state disgust and state fear, we examined correlations between these manipulation checks and the conformity index. Neither state disgust (*r* = -.04, *p* = .55) nor state fear (*r* = -.03, *p* = .64) was significantly correlated with conformity.

# **Supplementary Materials Study 1**

Text in square parenthesis was not presented to the participants.

Materials used for the manipulation

Participants were presented with images from the Culpepper Disgust Image Set. The image set is available via: Culpepper, P.D., Havlíček, J., Leongómez, J.D., & Roberts, S.C. (2018). Visually activating pathogen disgust: A new instrument for studying the behavioral immune system. *Frontiers in Psychology*, *9*, 1397. <https://doi.org/10.3389/fpsyg.2018.01397>

We used the corresponding pathogen-free images from the Image Set for the control condition.

Below are two examples of the images used.

| Treatment image: | Corresponding control image: |
| --- | --- |
| 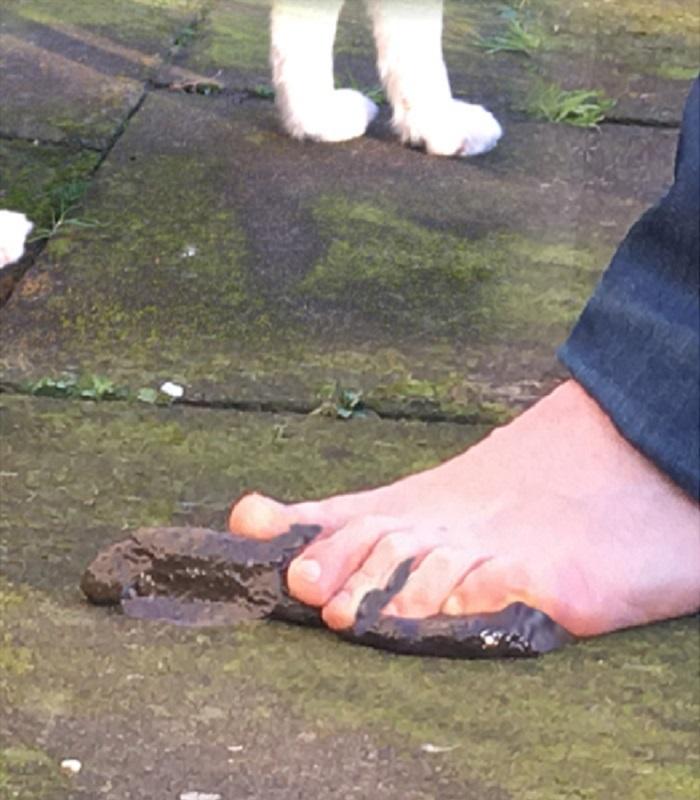 | 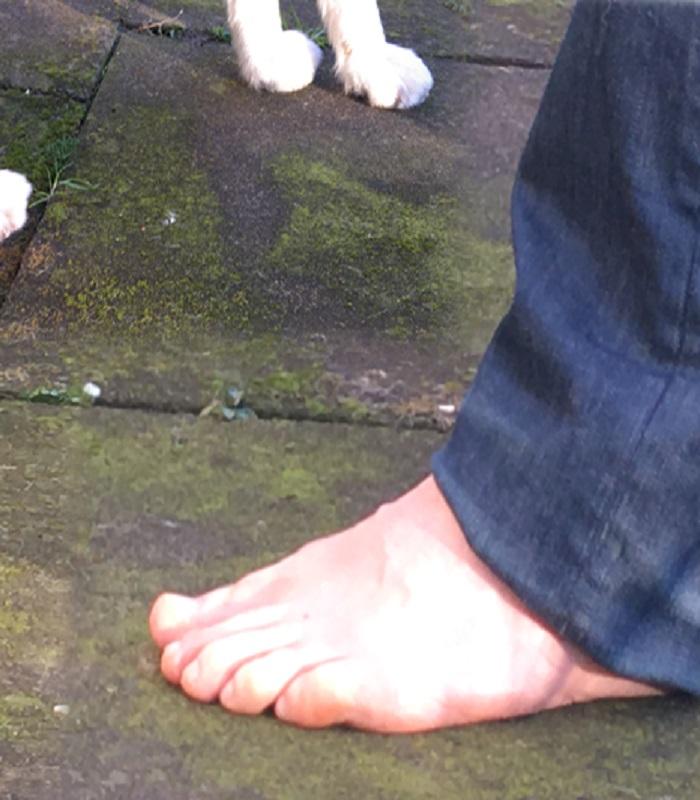 |

[Instruction to participants]

You will now see several photos for which you can indicate how you evaluate the photo.

[The selected images from the Culpepper Disgust Image Set (Culpepper et al., 2018) were presented one-by-one. Immediately below each image was the following question.]

How do you evaluate this photo?

| Very negative |  |  | Neutral |  |  | Very positive |
| --- | --- | --- | --- | --- | --- | --- |
| -3 | -2 | -1 | 0 | 1 | 2 | 3 |

State disgust

How much disgust do you experience at the moment?

- 0= No disgust (1)
- 1 (2)
- 2 (3)
- 3 (4)
- 4 (5)
- 5 (6)
- 6 (7)
- 7 (8)
- 8 (9)
- 9 (10)
- 10= Very much disgust (11)

Dependent variable

[Adherence to loyalty norms]

Below are several statements about you. Please read each statement and indicate how much you agree or disagree with that statement.

|  | Strongly disagree (1) | Disagree (2) | Somewhat disagree (3) | Neutral (4) | Somewhat agree (5) | Agree (6) | Strongly agree (7) |
| --- | --- | --- | --- | --- | --- | --- | --- |
| I act in ways that help my community. |  |  |  |  |  |  |  |
| In my actions, I try to show love for my country. |  |  |  |  |  |  |  |
| I help the members of my community. |  |  |  |  |  |  |  |
| I never betray my group. |  |  |  |  |  |  |  |
| I work to unite my community. |  |  |  |  |  |  |  |
| I always show loyalty. |  |  |  |  |  |  |  |
| I have an obligation to help members of my community. |  |  |  |  |  |  |  |
| I am proud of my country’s history. |  |  |  |  |  |  |  |
| It is important for me to play an active role in my community. |  |  |  |  |  |  |  |
| I am loyal to my family members. |  |  |  |  |  |  |  |
| I try to be a useful member of society. |  |  |  |  |  |  |  |
| It is more important for me to be a team player, than to express myself. |  |  |  |  |  |  |  |

[Adherence to hygiene norms]

Below are several statements about you. Please read each statement and indicate how much you agree or disagree with that statement.

[Note: The seventh answer option “strongly agree,” marked with an * below, was incorrectly translated to English. The Dutch version read “Helemaal mee eens” which indicates strong agreement. The incorrect English translation read “Strongly disagree”.]

|  | Strongly disagree (1) | Disagree (2) | Somewhat disagree (3) | Neutral (4) | Somewhat agree (5) | Agree (6) | Strongly agree (7)* |
| --- | --- | --- | --- | --- | --- | --- | --- |
| I always wash my hands after visiting a toilet. |  |  |  |  |  |  |  |
| When I go to the toilet, I always clean up after myself. |  |  |  |  |  |  |  |
| When I think I might be getting ill, I avoid physical contact with other people, so that I do not infect them. |  |  |  |  |  |  |  |
| I never sneeze in the direction of other people. |  |  |  |  |  |  |  |
| When I cook food for guests, I first wash my hands and then wash all the foods I will use. |  |  |  |  |  |  |  |
| When I go to the bathroom, I always wash my hands with water and soap. |  |  |  |  |  |  |  |
| When I use a public toilet, I make sure to clean up any mess I make. |  |  |  |  |  |  |  |
| When I cough in the presence of others, I always hold my hand in front of my mouth. |  |  |  |  |  |  |  |
| When I feel I have to sneeze, I always hold my hands in front of my mouth and nose. |  |  |  |  |  |  |  |
| When I prepare food for other people, I make sure I do everything hygienically. |  |  |  |  |  |  |  |

[Aversion to violations of loyalty norms]

Below are several statements. Imagine that each statement is said by a person of your age and sex. Then indicate how you would evaluate that person on the basis of these statements. Please try to imagine the situations as realistic as possible and answer the questions honestly and precisely. Please evaluate the situations independently of each other. To do that, judge each situation as if it is the only one you are evaluating. Please judge the person on a scale from “I would not view him/her negatively at all” to “I would view him/her very negatively”.

|  | I would not view him/her negatively at all 0 (1) | 1 (2) | 2 (3) | 3 (4) | 4 (5) | 5 (6) | I would view him/her very negatively 6 (7) |
| --- | --- | --- | --- | --- | --- | --- | --- |
| I do not act in ways that help my community. |  |  |  |  |  |  |  |
| In my actions, I try not to show love for my country. |  |  |  |  |  |  |  |
| I never help the members of my community. |  |  |  |  |  |  |  |
| I often betray my group. |  |  |  |  |  |  |  |
| I never work to unite my community. |  |  |  |  |  |  |  |
| I do not show loyalty. |  |  |  |  |  |  |  |
| I do not have an obligation to help members of my community. |  |  |  |  |  |  |  |
| I am not proud of my country’s history. |  |  |  |  |  |  |  |
| It is not important for me to play an active role in my community. |  |  |  |  |  |  |  |
| I am not loyal to my family members. |  |  |  |  |  |  |  |
| I never try to be a useful member of society. |  |  |  |  |  |  |  |
| It is less important for me to be a team player, than to express myself. |  |  |  |  |  |  |  |

[Aversion to violations of hygiene norms]

Below are several statements. Imagine that each statement is said by a person of your age and sex. Then indicate how you would evaluate that person on the basis of these statements. Please try to imagine the situations as realistic as possible and answer the questions honestly and precisely. Please evaluate the situations independently of each other. To do that, judge each situation as if it is the only one you are evaluating. Please judge the person on a scale from “I would not view him/her negatively at all” to “I would view him/her very negatively”.

|  | I would not view him/her negatively at all 0 (1) | 1 (2) | 2 (3) | 3 (4) | 4 (5) | 5 (6) | I would view him/her very negatively 6 (7) |
| --- | --- | --- | --- | --- | --- | --- | --- |
| I never wash my hands after visiting a toilet. |  |  |  |  |  |  |  |
| When I go to the toilet, I do not clean up after myself. |  |  |  |  |  |  |  |
| When I think I might be getting ill, I do not avoid physical contact with other people; I don’t care if I infect them. |  |  |  |  |  |  |  |
| I often sneeze in the direction of other people. |  |  |  |  |  |  |  |
| When I cook food for guests, I do not wash my hands and I do not wash the foods I will use. |  |  |  |  |  |  |  |
| When I go to the bathroom, I never wash my hands with water and soap. |  |  |  |  |  |  |  |
| When I use a public toilet, I never clean up the mess I make. |  |  |  |  |  |  |  |
| When I cough in the presence of others, I never hold my hand in front of my mouth. |  |  |  |  |  |  |  |
| When I feel I have to sneeze, I do not hold my hands in front of my mouth and nose. |  |  |  |  |  |  |  |
| When I prepare food for other people, I do not care much about doing it hygienically. |  |  |  |  |  |  |  |

Pathogen disgust sensitivity

Pathogen disgust sensitivity was measured with the instruction and items as described in Tybur et al. (2009).

[Instruction]

Please rate how disgusting you find the concepts described in the items below, where lower values mean that you find the concept less disgusting, and higher values mean that you find the concept more disgusting.

1. Shoplifting a candy bar from a convenience store
2. Hearing two strangers having sex
3. Stepping on dog poop
4. Stealing from a neighbor
5. Performing oral sex
6. Sitting next to someone who has red sores on their arm
7. A student cheating to get good grades
8. Watching a pornographic video
9. Shaking hands with a stranger who has sweaty palms
10. Deceiving a friend
11. Finding out that someone you don’t like has sexual fantasies about you
12. Seeing some mold on old leftovers in your refrigerator
13. Forging someone’s signature on a legal document
14. Bringing someone you just met back to your room to have sex
15. Standing close to a person who has body odor
16. Cutting to the front of a line to purchase the last few tickets to a show
17. A stranger of the opposite sex intentionally rubbing your thigh in an elevator
18. Seeing a cockroach run across the floor
19. Intentionally lying during a business transaction
20. Having anal sex with someone of the opposite sex
21. Accidentally touching a person’s bloody cut

| Not at all disgusting 0 | 1 | 2 | 3 | 4 | 5 | Extremely disgusting 6 |
| --- | --- | --- | --- | --- | --- | --- |

(The survey included other questions to measure demographics (age, sex) and variables unrelated to the current study.)

# **Supplementary Materials Study 2**

Text in square parenthesis was not presented to the participants.

Materials used in the manipulation

[Participants were shown eight images of White adult male faces taken from the Aging mind face database (Minear & Park, 2004). In the control condition, the images showed normal male faces. In the treatment condition the images were modified and showed the same eight faces, but with added salient pathogen cues. For each face, participants were asked two questions. All 16 images are available via the OSF page for this paper.]

How would you feel about shaking hands with the person in the picture?

- Very uncomfortable (1)
- Uncomfortable (2)
- Neutral (3)
- Comfortable (4)
- Very comfortable (5)

How healthy does this person look?

- Very unhealthy (1)
- Unhealthy (2)
- Neutral (3)
- Healthy (4)
- Very healthy (5)

Example of image used in control condition


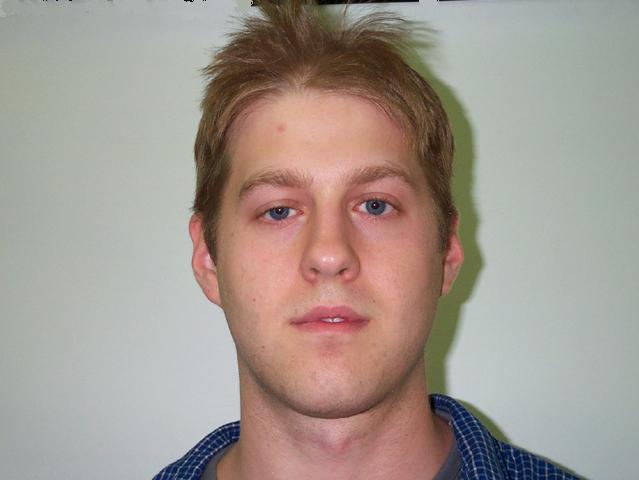


Example of image used in treatment condition


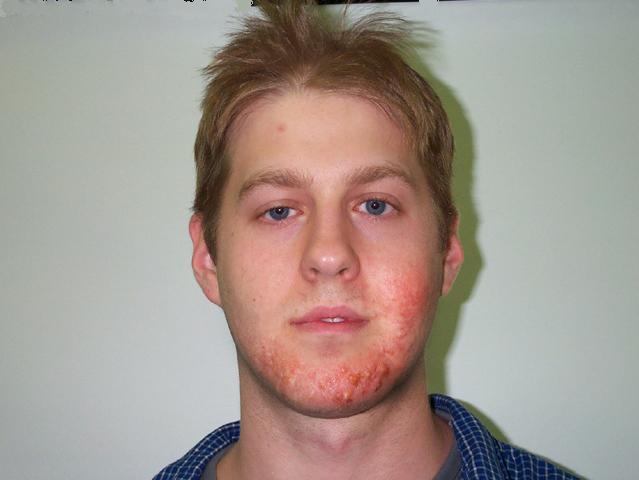


Dependent variable

The dependent variable was measured with the materials used in Study 1.

Pathogen disgust sensitivity

The same items were used as in Study 1. Items were rated on the scale shown below.

| Not at all disgusting 0 | 1 | 2 | 3 | 4 | 5 | Extremely disgusting 6 |
| --- | --- | --- | --- | --- | --- | --- |

The survey included other questions to measure demographics (age, sex) and variables unrelated to the current study.

# **Supplementary Materials Study 3**

Text in square parenthesis was not presented to the participants.

Measure Dutch nationality

Do you have the Dutch nationality (*Nederlandse nationaliteit*)? Yes (1), No (2), Prefer not to say (3)

Materials used in the manipulation

[The manipulation was similar to that used in Study 2. Participants were shown eight images of White adult male faces taken from the Aging mind face database (Minear & Park, 2004). In the control condition, the images showed normal male faces. In the treatment condition the images were modified and showed the same eight faces, but with added salient pathogen cues. For each face, participants were asked three questions.]

How would you feel about shaking hands with the man in the photo?

Answered with an 11-point slider with anchors: very uncomfortable (-5), neutral (0), very comfortable (5)

How would you feel about this man sitting next to you on the bus?

Answered with an 11-point slider with anchors: very uncomfortable (-5), neutral (0), very comfortable (5)

Does this man look ill or healthy?
Answered with an 11-point slider with anchors: very ill (-5), neutral (0), very healthy (5)

Dependent variable

[The dependent variable consisted of responses to the writers essays that contained anti- vs. pro-ingroup statements. After reading each essay the participants answered 7 questions that measure attitudes toward the writer of the essay. The dependent variable is the average evaluation of the writer of the pro-ingroup essay. This dependent variable was modeled on the one used by Navarrete & Fessler (2006). First the participants were presented with the anti-ingroup essay.]

Below is an opinion of the Netherlands posted somewhere on the internet.
  *When I first came to the Netherlands from my home country, I believed it was the “land of opportunity” but I soon realized this was only true for the rich. The system here is set up for rich against the poor. All people care about here is money and trying to have more than other people. This no sympathy for people. Its all one group putting down others and nobody cares about the foreigners. The people only let foreigners have jobs like pick fruit or wash dishes because no Dutch people would do it. Dutch people are spoiled and lazy and want everything handed to them. Holland is a cold country that is unsensitive to needs and problems of foreigners. It thinks it’s a great country but its not.*

How likeable is the author of this opinion?

|  | Not at all likeable | Neutral | Very likeable |
| --- | --- | --- | --- |

|  | -4 | -3 | -2 | -1 | 0 | 1 | 2 | 3 | 4 |
| --- | --- | --- | --- | --- | --- | --- | --- | --- | --- |

| 1 () | 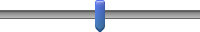 |
| --- | --- |

How intelligent is the author?

|  | Very unintelligent | Neutral | Very intelligent |
| --- | --- | --- | --- |

|  | -4 | -3 | -2 | -1 | 0 | 1 | 2 | 3 | 4 |
| --- | --- | --- | --- | --- | --- | --- | --- | --- | --- |

| 1 () | 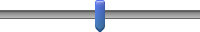 |
| --- | --- |

How knowledgeable is the author?

|  | Not at all knowledgeable | Neutral | Very knowledgeable |
| --- | --- | --- | --- |

|  | -4 | -3 | -2 | -1 | 0 | 1 | 2 | 3 | 4 |
| --- | --- | --- | --- | --- | --- | --- | --- | --- | --- |

| 1 () | 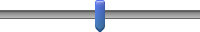 |
| --- | --- |

How moral is the author?

|  | Very immoral | Neutral | Very moral |
| --- | --- | --- | --- |

|  | -4 | -3 | -2 | -1 | 0 | 1 | 2 | 3 | 4 |
| --- | --- | --- | --- | --- | --- | --- | --- | --- | --- |

| 1 () | 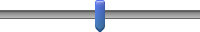 |
| --- | --- |

How mentally well-adjusted is the author?

|  | Not at all adjusted | Neutral | Very adjusted |
| --- | --- | --- | --- |

|  | -4 | -3 | -2 | -1 | 0 | 1 | 2 | 3 | 4 |
| --- | --- | --- | --- | --- | --- | --- | --- | --- | --- |

| 1 () | 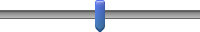 |
| --- | --- |

How truthful is the author?

|  | Not at all truthful | Neutral | Very truthful |
| --- | --- | --- | --- |

|  | -4 | -3 | -2 | -1 | 0 | 1 | 2 | 3 | 4 |
| --- | --- | --- | --- | --- | --- | --- | --- | --- | --- |

| 1 () | 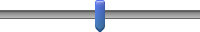 |
| --- | --- |

How much would you want to work together with the author?

|  | I would definitely not like to work with the author | Neutral | I would definitely like to work with the author |
| --- | --- | --- | --- |

|  | -4 | -3 | -2 | -1 | 0 | 1 | 2 | 3 | 4 |
| --- | --- | --- | --- | --- | --- | --- | --- | --- | --- |

| 1 () | 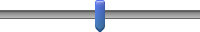 |
| --- | --- |

[On the next page of the survey the participants were presented with the pro-ingroup essay.]

Below is another opinion of the Netherlands posted somewhere on the internet.
*The most important thing about the Netherlands is the incredible freedom people have.  In other countries, everything is not as good. Here there is freedom to go to school, freedom to work in any job you want.  In this country people can go to school and train for the job they want.  Here anyone who works hard can make their own success.  In other countries most people live in poverty with no chance of escape.  In this country people have more opportunity for success than in any other and success does not depend on the group you belong to. While there are problems in any country, the Netherlands truly is a great nation and I am happy to be a Dutch citizen.*

How likeable is the author of this opinion?

|  | Not at all likeable | Neutral | Very likeable |
| --- | --- | --- | --- |

|  | -4 | -3 | -2 | -1 | 0 | 1 | 2 | 3 | 4 |
| --- | --- | --- | --- | --- | --- | --- | --- | --- | --- |

| 1 () | 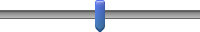 |
| --- | --- |

How intelligent is the author?

|  | Very unintelligent | Neutral | Very intelligent |
| --- | --- | --- | --- |

|  | -4 | -3 | -2 | -1 | 0 | 1 | 2 | 3 | 4 |
| --- | --- | --- | --- | --- | --- | --- | --- | --- | --- |

| 1 () | 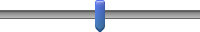 |
| --- | --- |

How knowledgeable is the author?

|  | Not at all knowledgeable | Neutral | Very knowledgeable |
| --- | --- | --- | --- |

|  | -4 | -3 | -2 | -1 | 0 | 1 | 2 | 3 | 4 |
| --- | --- | --- | --- | --- | --- | --- | --- | --- | --- |

| 1 () | 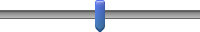 |
| --- | --- |

How moral is the author?

|  | Very immoral | Neutral | Very moral |
| --- | --- | --- | --- |

|  | -4 | -3 | -2 | -1 | 0 | 1 | 2 | 3 | 4 |
| --- | --- | --- | --- | --- | --- | --- | --- | --- | --- |

| 1 () | 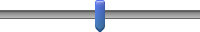 |
| --- | --- |

How mentally well-adjusted is the author?

|  | Not at all adjusted | Neutral | Very adjusted |
| --- | --- | --- | --- |

|  | -4 | -3 | -2 | -1 | 0 | 1 | 2 | 3 | 4 |
| --- | --- | --- | --- | --- | --- | --- | --- | --- | --- |

| 1 () | 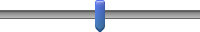 |
| --- | --- |

How truthful is the author?

|  | Not at all truthful | Neutral | Very truthful |
| --- | --- | --- | --- |

|  | -4 | -3 | -2 | -1 | 0 | 1 | 2 | 3 | 4 |
| --- | --- | --- | --- | --- | --- | --- | --- | --- | --- |

| 1 () | 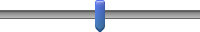 |
| --- | --- |

How much would you want to work together with the author?

|  | I would definitely not like to work with the author | Neutral | I would definitely like to work with the author |
| --- | --- | --- | --- |

|  | -4 | -3 | -2 | -1 | 0 | 1 | 2 | 3 | 4 |
| --- | --- | --- | --- | --- | --- | --- | --- | --- | --- |

| 1 () | 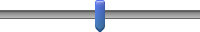 |
| --- | --- |

Pathogen disgust sensitivity

Pathogen disgust sensitivity was measured with the same items as in Study 1. All items were rated on the 11-point scale shown below.

|  | Not disgusting at all | Neutral | Extremely disgusting |
| --- | --- | --- | --- |

|  | 0 | 1 | 2 | 3 | 4 | 5 | 6 | 7 | 8 | 9 | 10 |
| --- | --- | --- | --- | --- | --- | --- | --- | --- | --- | --- | --- |

| () | 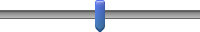 |
| --- | --- |

The survey included other questions to measure demographics (age, sex) and variables unrelated to the current study.

# **Supplementary Materials Study 4**

Text in square parenthesis was not presented to the participants.

Materials used to manipulate perceived infection risk

Images used were from the Culpepper Disgust Image Set available via: Culpepper, P.D., Havlíček, J., Leongómez, J.D., & Roberts, S.C. (2018). Visually activating pathogen disgust: A new instrument for studying the behavioral immune system. *Frontiers in Psychology*, *9*, 1397. <https://doi.org/10.3389/fpsyg.2018.01397>

[Instruction]

Please look carefully at the 10 photo's on the following pages. When you are looking at the photo's, a button will appear below the photo after 4 seconds. Click the button to proceed to the next page.

Dependent variable

The next part of the research is about the evaluation of modern art.

On the following pages you will rate 10 paintings on a 10-point scale from "not at all beautiful" to "very beautiful". These paintings have previously been evaluated in a study among students at Tilburg University. Below each painting you will find the average rating of the students in the previous study.

[On the next 10 pages participants were presented with the image of a painting, with immediately below shown the average rating of other students. See below. The 10 images were taken from the Internet. One example is shown below. Participants rated all 10 images on the scale shown below. The average ratings of other students were shown as: 5.4, 6.5, 6.6, 5.1, 7.4, 5.1, 3.8, 5.2, 5.1, 5.1.]


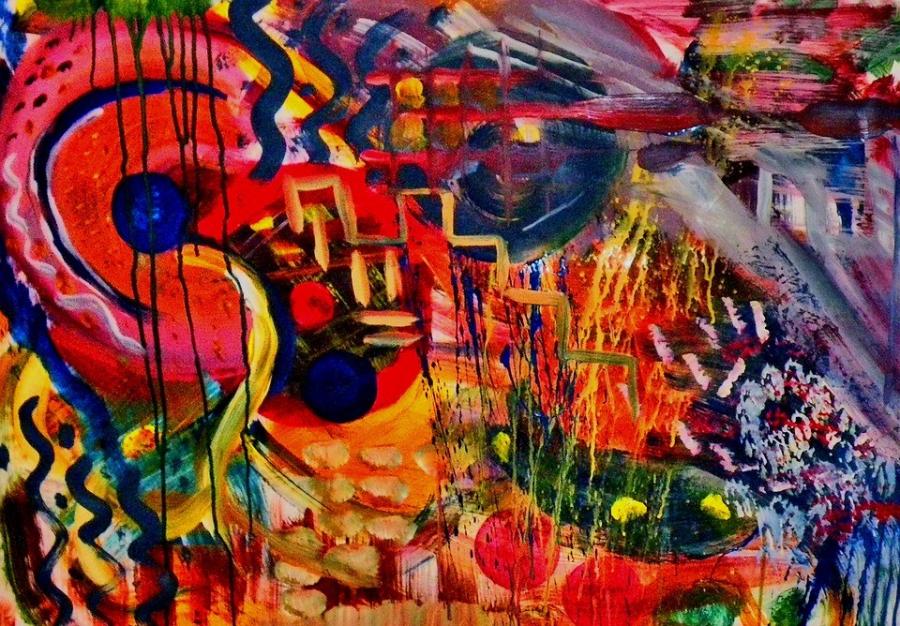


*​Average rating of Tilburg University students = 5.4*

|  | Not at all beautiful = 1 (1) | 2 (2) | 3 (3) | 4 (4) | 5 (5) | 6 (6) | 7 (7) | 8 (8) | 9 (9) | Very beautiful = 10 (10) |
| --- | --- | --- | --- | --- | --- | --- | --- | --- | --- | --- |
| 1 (1) |  |  |  |  |  |  |  |  |  |  |

Mood and emotions

[Following Wu & Chang (2012) we included measures of mood and emotions.]

Please indicate how you are feeling right now.

- very unpleasant (1)
- unpleasant (2)
- neutral (3)
- pleasant (4)
- very pleasant (5)

Please indicate how you are feeling right now.

- very bad (1)
- bad (2)
- neutral (3)
- good (4)
- very good (5)

Please indicate how you are feeling right now.

- very unhappy (1)
- unhappy (2)
- neutral (3)
- happy (4)
- very happy (5)

[On the next page]

Below are a number of feelings and emotions. Indicate to what extent you are currently experiencing these feelings and emotions. 0 means that you do not experience it at, and 6 means that you experience it very much.

|  | Not at all = 0 (1) | 1 (2) | 2 (3) | 3 (4) | 4 (5) | 5 (6) | Very much= 6 (7) |
| --- | --- | --- | --- | --- | --- | --- | --- |
| Uneasy |  |  |  |  |  |  |  |
| Anxious |  |  |  |  |  |  |  |
| Worried |  |  |  |  |  |  |  |
| Grossed out |  |  |  |  |  |  |  |
| Disgusted |  |  |  |  |  |  |  |
| Nauseated |  |  |  |  |  |  |  |

Secondary measure of conformity: Conformity to the vacation group

Imagine that you will go on vacation with a group of friends this summer. Because you already have to spend a lot of money on the trip, you all have agreed to look for a cheap accommodation. Last weekend your friends found a cheap holiday home that they would like to reserve. The location of the holiday home is good and the price is within budget. Everyone as already agreed to the choice, except you. Your friends have asked if you also agree with the reservation of this holiday home.

Before you agree, you search the internet for information about the holiday home. On the internet you find some pictures of the holiday home, below you can see these pictures. The pictures show that the holiday home has a filthy bathroom with mold.


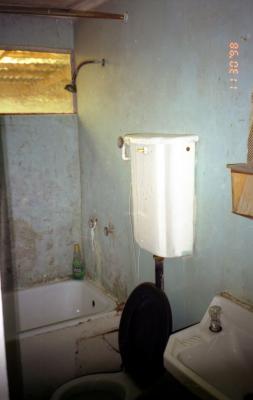

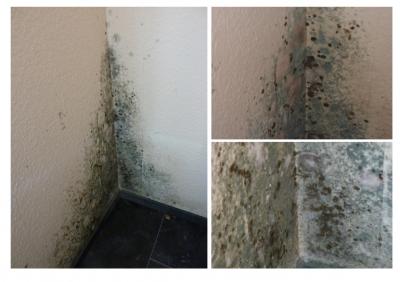


Below are some statements about how you might respond to your friends. Indicate for each statement to what extent you agree or disagree.

|  | Strongly disagree (1) | Disagree (2) | Somewhat disagree (3) | Neutral (4) | Somewhat agree (5) | Agree (6) | Strongly agree (7) |
| --- | --- | --- | --- | --- | --- | --- | --- |
| I would certainly approve of the group's choice for this holiday home |  |  |  |  |  |  |  |
| I would never accept the choice of this holiday home |  |  |  |  |  |  |  |
| Although the chosen holiday home is not optimal, I would adjust to the choice of the group. |  |  |  |  |  |  |  |
| If my friends want to go to such a filthy holiday home, I'd rather not go. |  |  |  |  |  |  |  |

Pathogen disgust sensitivity

Pathogen disgust sensitivity was measured with the same items as in Study 1. All items were rated on the scale shown below.

| Not at all disgusting 0 | 1 | 2 | 3 | 4 | 5 | Extremely disgusting 6 |
| --- | --- | --- | --- | --- | --- | --- |

The survey included other questions to measure demographics (age, sex) and variables unrelated to the current study.

# **Supplementary Materials Study 5**

## **Method**

### ***Procedure***

The study involved an online survey. The study included a between-subjects design with two conditions. Participants were randomly assigned to one of two conditions: a Disease threat condition and a Neutral control condition. In the Disease threat condition, participants completed a guided-recall task that was designed to evoke memories of exposure to infectious disease (see details below). In the Neutral control condition, participants completed a guided-recall task that was designed to *not* evoke memories of a particular threat; participants were asked to recall what they did on the previous day. The experiment of Murray & Schaller (2012) also included a third “Other threat” condition in which a non-disease related threat was made salient. However, their study did not find any difference in conformity between the Other threat condition and the Neutral control condition. The primary goal of the current experiment is to test whether an increase in disease threat increases conformity, compared to a neutral control condition. Therefore, the current experiment did not include an Other threat condition.

In Murray & Schaller (2012), the guided-recall asked involved a face-to-face interview with the experimenter after which participants completed some of the dependent variables. In order to maintain the salience of the disease threat, after approximately 10 minutes participants were given an additional reminder: participants were asked to write down the events they had described to the experimenter. After this reminder, participants completed the remaining dependent variables. In the current experiment, the guided-recall task used the same questions, but asked participants to type their answers to the questions. Given the reduced number of dependent variables in the current study, no reminder was used.

The guided-recall task that we used was modeled on the task used by Murray & Schaller (2012), with adjustments to make the procedure suitable for an online survey. There are numerous studies that have used guided-recall by asking participants to write about an event (without a face-to-face interview; e.g., Lerner & Keltner, 2001; Dunn & Schweitzer, 2005; Whitson & Galinsky, 2008). This suggests that the guided-recall procedure is amenable to an online study. In the Disease threat condition, participants were instructed to take approximately 5 minutes, recall some experiences from their own lives, and describe their experiences (i.e., by writing text in a text box). Participants were asked to describe (1) how they typically feel when they come in contact with germs, (2) how they typically feel when they are forced to be close to someone who you think is sick or diseased, (3) how they felt during a specific time in their life that they felt especially vulnerable to germs, and (4) how they felt during a time when they had to come into close contact with a person who was sick or diseased. In the control condition, participants were asked to recall what they did the day before, to describe any new stimuli they came in contact with, what thoughts this activated, and how this made them feel. Below are the full instructions for the Pathogen threat condition:

For this part of the experiment, we will be asking you to discuss your reactions to some events, and asking you to give some examples. The objective of this task is for you to recall freely the events that occurred, and the feelings and physical reactions that you had to these events. The point of this exercise is not to simply answer the questions that we ask you, but rather to most vividly recall your experience. This task will include four questions and will take approximately 5 minutes.

[page break]

1. Please take a moment to think **how you typically feel when you come into contact with germs**. In the textbox below, describe your typical emotional and physical reactions when you come into contact with germs. (After one minute the button will appear and you can proceed to the next page.)

[textbox]

[page break]

2. Please take a moment to think **how you typically feel when you are forced to be close to someone who you think is sick or diseased**. In the textbox below, describe your emotional and physical reactions. (After one minute the button will appear and you can proceed to the next page.)

[textbox]

[page break]

3. Now, can you think of **a specific time in your life that you felt especially vulnerable to germs**? For example, can you recall a time that you were in a hospital, or a time that you touched something really dirty, or a time that you felt that you may have eaten or come into contact with an object that carries disease? In the textbox below, describe

- 1. what it was about this situation that made you feel threatened.
  2. the specific stimuli that made you mindful of this threat.
  3. the emotions that you were feeling.
  4. the thoughts that were running through your head.
  5. how your body felt (For example, did you feel tight in some areas? Tense? Sweating?)

(After one minute the button will appear and you can proceed to the next page.)

[textbox]

[page break]

4. Can you think of **a time when you had to come into close contact with a person who was sick or diseased**? If you already described such a situation at the previous question, then please try to think of another time you had to come in close contact with a person who was sick or diseased. In the textbox below, describe

1. what it was about this situation that made you feel threatened.
2. the specific stimuli that made you mindful of this threat.
3. the emotions that you were feeling
4. the thoughts that were running through your head
5. how your body felt (For example, did you feel tight in some areas? Tense? Sweating?)

(After one minute the button will appear and you can proceed to the next page.)

[textbox]

[page break]

Below are the full instructions for the control condition:

For this part of the experiment, we will be asking you to recall a time in your past. The objective of this task is for you to recall freely the events that occurred, and the feelings and physical reactions that you had to these events. The point of this exercise is not to simply answer the questions that we ask you, but rather to most vividly recall your experience. This task will include five questions and will take approximately 5 minutes.

1. Please take a moment to recall what you did yesterday. Describe how you were feeling throughout the day. (After one minute the button will appear and you can proceed to the next page.)

[textbox]

[page break]

2. Describe any novel stimuli that you may have come into contact with. (After one minute the button will appear and you can proceed to the next page.)

[textbox]

[page break]

3. Describe the emotions that you were feeling. (After one minute the button will appear and you can proceed to the next page.)

[textbox]

[Page break]

4. Describe any special thoughts that were running through your head. (After one minute the button will appear and you can proceed to the next page.)

[textbox]

[Page break]

5. Describe how your body felt. (For example, did you feel tight in some areas? Tense? Sweating?) (After one minute the button will appear and you can proceed to the next page.)

[textbox]

[Page break]

After the guided-recall task, participants completed four measures of conformity, three of which—self-reported conformist attitudes, liking for people with conformist traits, valuation of obedience—were the same as those used by Murray & Schaller (2012). The fourth measure was similar but not identical to the behavioral measure used by Murray & Schaller (2012). Originally, the behavioral measure asked participants (who were university students) to indicate their agreement or disagreement with a proposal about an education policy at their university. They could indicate their agreement by putting a coin into one of two cups (labeled AGREE and DISAGREE), with always one of the cups containing only 3 coins and the other containing 25 coins, thus indicating whether the majority agreed or disagreed with the policy. Conformity was operationalized as putting their coin in the cup with the majority of coins. In order to adjust this behavioral measure to an online context, we asked people whether they agreed or disagreed with the statement “The National Science Foundation should increase its budget for research of the biodiversity in Antarctica.” The instructions mentioned that in a survey one year prior we had also asked about this issue and that this previous survey indicated that a majority (88%) agreed (or disagreed, randomized across participants) with the statement. The full instructions read:

Some people have proposed that the National Science Foundation increases its budget for research of the biodiversity in Antarctica. Last year we asked a sample of Americans via Prolific what they think about this issue. We now collect opinions on this issue again, so that we can see if public opinion on this issue has changed or stayed the same.

Please indicate whether you agree or disagree with the statement below.

*The National Science Foundation should increase its budget for research of the biodiversity in Antarctica.*

Results from the previous survey:

Agree: 88% of respondents

Disagree: 12% of respondents

[Buttons to answer Agree or Disagree]

Conformity was indicated by selecting the majority opinion. The order of the four measures of conformity was randomized across participants.

Participants then completed a measure of the emotions they experienced during the experimental session, each emotion rated on a 7-point scale from *None at all* (1) to *Extremely* (7), including a measure of state-disgust that served as a manipulation check. Finally, participants reported their political orientation and completed the seven items of the pathogen disgust domain of the TDDS. The survey also included an attention check. Immediately after the last item for pathogen disgust sensitivity, participants were shown the item *Please select ‘not at all disgusting’*, with the same seven answer options as for the TDDS. Participants who selected any other answer than “not at all disgusting” were considered to have failed the attention check. Finally, participants were thanked and debriefed.

### ***Participants***

Following the pre-registration, we collected data in batches from USA residents via Prolific. Batch 1 included 280 participants and if the manipulation checks would indicate that the manipulation works as intended, then we would proceed with collecting 508 participants in a second batch. We aimed to recruit in total 788 participants (394 participants per condition) because assuming a population effect size of d=0.20 (which is smaller than that observed in the original study), the required sample size for a for a two-sided t-test for independent means with 80% power is at least 788 (394 per condition). This sample size would also allow for a small-telescopes analysis (which in this case would require N=358).

The purpose of batch 1 was to assess whether the Disease threat manipulation was successful in increasing disease threat. To do so, we made two comparisons (with one-sided t-tests) of the means of state disgust and state fear: (1) testing if state disgust is higher (with p ≤ .05 and Cohen's d ≥ 0.3) in the Disease threat condition than in the Neutral condition and (2) testing if in the Disease threat condition, state disgust is higher (with p ≤ .05 and Cohen's d ≥ 0.3) than state fear. We consider the manipulation successful when there is evidence that (a) state disgust is higher in the Disease threat condition than in the Neutral condition and (b) in the Disease threat condition state disgust is higher than state fear. We recruited 280 participants in batch 1 because for this sample size, the power for each of these tests (assuming d=0.3) is .80 and .99, respectively.

## **References with Study 5**

Dunn, J. R., & Schweitzer, M. E. (2005). Feeling and Believing: The Influence of Emotion on Trust. *Journal of personality and social psychology*, *88*(5), 736-748.

Lerner, J. S., & Keltner, D. (2001). Fear, anger, and risk. *Journal of personality and social psychology*, *81*(1), 146-159.

Whitson, J. A., & Galinsky, A. D. (2008). Lacking control increases illusory pattern perception. *Science*, *322*(5898), 115-117.
